# Developing an inclusive research (methods) strategy for the Manchester Biomedical Research Centre and Clinical Research Facility

**DOI:** 10.1371/journal.pone.0357608

**Published:** 2026-09-16

**Authors:** Anna Coleman, Sheela Medahunsi, Joanne Elliott, Jedidah Mould, Abin Thomas, Eliza Varga, Arpana Verma

**Affiliations:** 1 Inclusive Research Methods Team, NIHR Manchester Biomedical Research Centre and Clinical Research Facility, Division of Medical Education, School of Medical Sciences, Faculty of Biology, Medicine and Health, University of Manchester, United Kingdom; 2 Manchester City Council, United Kingdom; Public Health England, UNITED KINGDOM OF GREAT BRITAIN AND NORTHERN IRELAND

## Abstract

As funders of health and care research increasingly mandate inclusive research practices, the Manchester Biomedical Research Centre (BRC) and Clinical Research Facility (CRF) developed a comprehensive Inclusive Research (IR) Strategy to embed the principles of inclusivity across their experimental medicine research. This paper presents the co-production of the Strategy, shaped by collaboration between Inclusive Research Methods (IRM), Equality, Diversity and Inclusion (EDI), and Patient and Public Involvement and Engagement (PPIE) teams, alongside public contributors and wider stakeholders. The Strategy development was informed by iterative engagement, including workshops and one-to-one consultations, which revealed varying levels of understanding of IR and highlighted the need for accessible, evidence-informed approaches. It is structured around five strategic principles: mapping inequalities and protected characteristics; training and capacity building; working with communities and partners; establishing effective routes to delivery and using and creating Inclusive Research Methods. Key learning from the process includes the importance of aligning IR with existing organisational strategies, fostering inclusive governance through our Inclusive Research Oversight Board (IROB), and ensuring flexibility to adapt to emerging national guidance—such as National Institute for Health and Care Research’s (NIHR) 10-step framework for inclusive research design or Core20PLUS5 – and the ongoing cycles of evaluation across the BRC/CRF programmes. Stakeholder feedback also underscored the value of co-production, the need to dismantle power dynamics, and the importance of clarity, accountability, and responsiveness in strategic implementation. The IR Strategy demonstrates how inclusive research can be operationalised within a complex translational research infrastructure. It offers a replicable model for embedding inclusive practices across the research lifecycle, leading to more equitable participant recruitment, enhanced research relevance, and health research with greater impact for all, including under-served populations. By fostering team research and an inclusive culture where inclusivity is business as usual, the IR Strategy seeks to drive meaningful change in health and care research, ensuring it is representative, accessible, and beneficial to all over the longer term.

## Introduction

As expectations for Inclusive Research (IR) move from aspiration to a condition of funding [1,2], research infrastructures face a critical challenge: how to translate principles of inclusion into sustainable organisational practice, research culture, and methodological innovation. In the UK, Biomedical Research Centres (BRCs) and Clinical Research Facilities (CRFs) are increasingly expected to demonstrate that inclusivity is embedded throughout the research lifecycle, yet few published examples describe how this can be achieved in practice at an organisational level. This paper describes how BRC/CRF Manchester responded to this challenge by establishing a dedicated Inclusive Research Methods (IRM) function, governance structure and Strategy. This has helped to embed inclusivity across research design, delivery, culture and organisational systems, addressing concerns about the relevance of scientific outputs as a key aspect of responsible research conduct, with attention to both process and societal benefit [3].

This challenge is particularly acute in experimental medicine, where studies may involve early-phase interventions, intensive procedures, biomarker-led eligibility, specialist facilities, or tightly defined inclusion and exclusion criteria. These features can strengthen internal validity and participant safety but may also narrow who is able or invited to take part. Embedding IR in this context therefore requires careful attention to both scientific rigour and equitable access, ensuring that methodological decisions designed to protect study quality do not inadvertently reproduce patterns of exclusion or limit the relevance of findings for populations most affected by the conditions under investigation.

In autumn 2024, the National Institute for Health and Care Research (NIHR) declared inclusion a core operating principle, stating ‘Good research must include people from the diverse populations of the UK and include more women, children and older people’. NIHR emphasised that IR is essential to improving health and care for all and reducing inequalities [2]. It defined IR as being ‘intentionally designed, conducted and communicated inclusively, producing rigorous, generalisable, impactful science that benefits the entire population’. Improving diversity, equity and inclusion in research is a shared responsibility, critical to enhancing scientific rigor and accuracy [4].

A recent study [5] highlighted that while the exclusion of certain population groups from research may be unintentional, it can nonetheless undermine the generalisability, quality, relevance, and integrity of research outcomes. Addressing this issue is crucial, and Yu et al [6] emphasise the importance of ensuring adequate representation to enable the development of effective interventions that benefit the entire population. Furthermore, the NIHR [7] stresses that IR practices are essential to ensure that individuals from marginalised communities have equal access to evidence-based care.

These ideas are also being enacted in the policy arena. For example, the National Healthcare Inequalities Improvement Programme [8] exemplifies this shift, working across the National Health Service (NHS) in England, health and care system partners, patients and communities, to deliver equitable high-quality healthcare.

In this context, this paper outlines the development of an IR Strategy for BRC/ CRF Manchester designed. The work represents an innovative approach within BRC and CRF infrastructure, complimenting existing Equality, Diversity and Inclusion (EDI) and Patient and Public Involvement and Engagement (PPIE) functions, to establish IRM as a distinct strategic function with dedicated governance, expertise and evaluation mechanisms. Developed prior to NIHR’s introduction of IR design as a formal funding requirement [2], the Strategy provides a practical, evidence-informed framework for operationalising inclusivity across the research lifecycle. It was developed as part of Manchester BRC/CRF’s strategic commitment to embedding research inclusivity through dedicated methods, governance and organisational change. By describing the processes and lessons learned from developing and implementing an IR Strategy, this paper offers a potentially transferable model for other BRCs, CRFs and research organisations seeking to embed IR systematically and at scale.

### Research inclusivity and the Manchester research / healthcare context

#### Research inclusivity.

To enhance research inclusivity, deliberate action is required to ensure that everyone feels included, respected, and valued throughout every stage of the research process, from planning to dissemination. This is important for many reasons including improving science (rigor, accuracy and generalisability), reducing inequalities, increasing relevance of the research, improving safety, enhancing economic benefits and fulfilling a moral imperative to benefit all. Within experimental medicine, these benefits are especially important because early translational studies can shape downstream evidence generation, clinical pathways and innovation pipelines; exclusions at this stage may therefore have consequences that extend beyond a single study.

A paradigm shift can be seen in the way research is conceptualised and conducted when considering research inclusivity. This marks a transition from conducting research on people to conducting research with them [9, p278]. Initially rooted in the field of learning disability [10] the concept has gradually gained traction across disciplines, albeit at varying rates.

More recently, the need for researchers to recognise and address biases that compromise the validity and relevance of research has been emphasised [4]. Enhancing the representativeness of research samples is essential to improving generalisability and ensuring findings benefit the widest possible range of populations. IR is often reduced to a matter of compliance, something to be documented or satisfied. However, such a framing obscure more substantive questions about whose knowledge is valued, how agendas are set, partnerships formed, and research is communicated and credited. At the same time, increasing pressures on researchers’ time and the fragmentation of guidance across policies, toolkits and funding bodies make it challenging to engage meaningfully with these issues, even where there is clear intent to do so.

#### Manchester (BRC/ CRF) infrastructure.

The Manchester BRC and CRF operate within a nationally funded infrastructure designed to accelerate translational research and improve health outcomes. Funded by the NIHR over a five-year cycle, BRCs are strategic partnerships between NHS organisations and Universities that aim to convert scientific discoveries into new treatments, diagnostics, and technologies.

In 2022, the Manchester BRC was awarded £64.1 million to expand its research across Greater Manchester, Lancashire and South Cumbria. This investment supports a broader geographical reach and thematic scope than the previous BRC, enabling it to address health inequalities across diverse urban, rural and coastal communities.

The Manchester CRF complements this mission by providing dedicated facilities for experimental medicine research. It conducts both commercial and non-commercial studies. The CRF works with patients, hospitals, universities and industry to transform scientific breakthroughs into diagnostic tests and life-saving treatments for patients driving personalised health and care for all. The Strategic aims of both programmes are shown in Table 1.

**Table 1:** The Strategic aims of Manchester BRC and CRF.

*Manchester BRC* [11,12]	*Manchester CRF* [12]
**“Embed** early translational research further into our communities and localities by deepening the meaningful involvement of patients, public and civic partners.	**“Expand CRF** in response to the needs of GM’s population and the national life science industry to expand the volume and breadth of world-class experimental medicine research as part of an integrated GM approach.
**Build** a unique national powerhouse for innovation by combining our partnership’s world-leading discovery and translational science capabilities with a strong research culture centred on a committed, diverse and inclusive workforce.	**Increase the skills and capabilities of the workforce** to safely deliver complex experimental medicine studies through provision of tailored training and educational programmes.
**Accelerate** at scale, the impact of our research through our mature and integrated innovation pipeline to achieve measurable improvements in health and wellbeing across all sections of society in our region and beyond”	**Involve patients and the public** in the co-production of systems to facilitate engagement and widen opportunities to access experimental medicine studies **focussing on under-served populations”**.

The CRF context also illustrates why IR must be operational as well as aspirational. Experimental medicine studies often require participants to attend specialist facilities, undergo multiple visits or procedures, meet narrowly defined eligibility criteria, and navigate complex information about risk, uncertainty and potential benefit. These requirements can create practical, linguistic, financial, cultural and trust-related barriers for some groups, particularly those already under-served by health research. Conversely, when inclusive approaches are built into feasibility assessment, site selection, recruitment planning, consent processes and participant support, experimental medicine can become more accessible, acceptable and relevant to the communities it ultimately aims to benefit.

**Fig 1:**
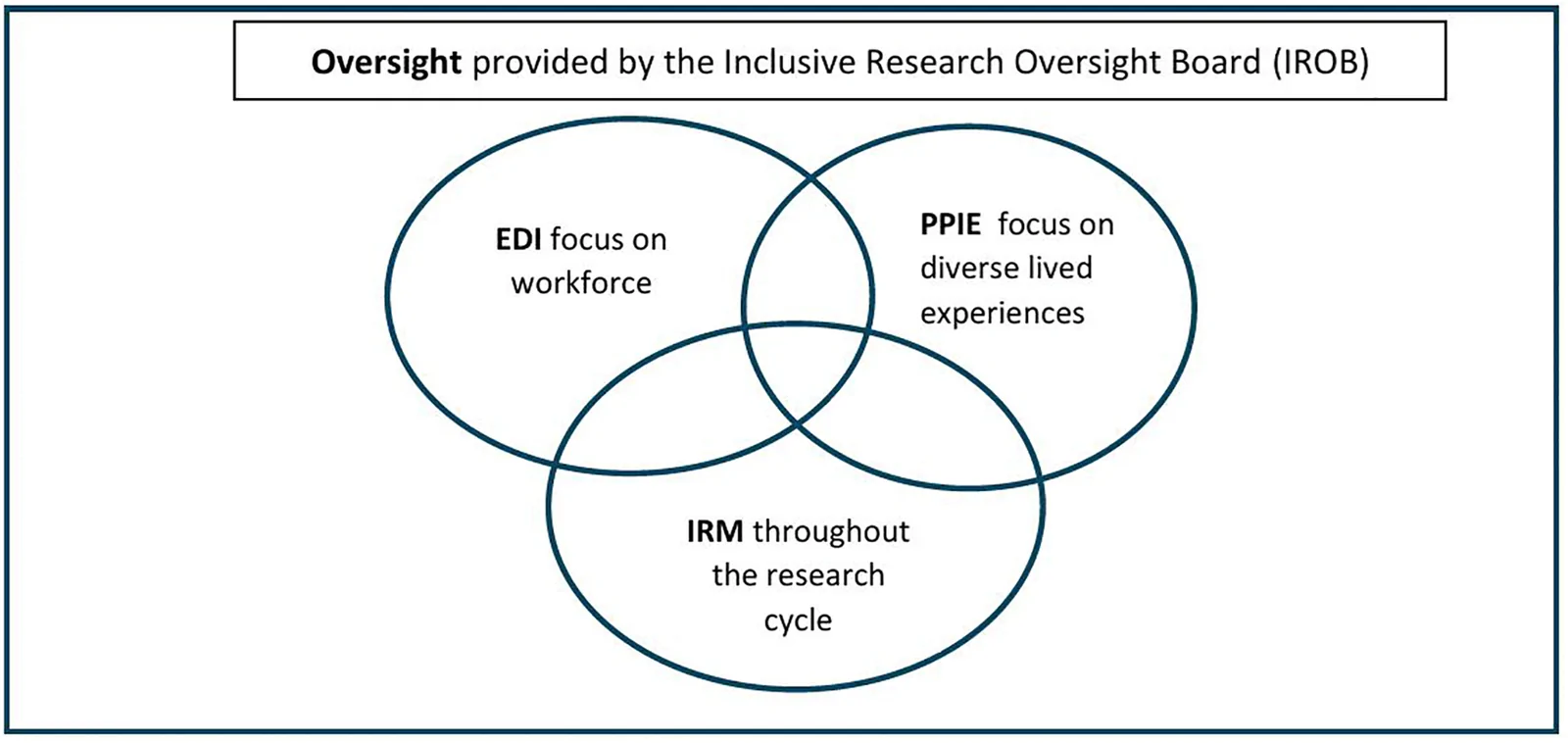
Research inclusivity structure for Manchester BRC/ CRF.

This is the second round of NIHR funding for Manchester BRC/ CRF (2022–2027), and during the initial funding period, inclusivity efforts were primarily channelled through a Health Inequalities Steering Group and the combined functions of EDI and PPIE. Based on recommendations from the BRC/CRF external Scientific Advisory Board, (research) inclusivity was restructured into three distinct functions: EDI, PPIE and IRM for the second round of funding. Each function is supported by a small, dedicated team and overseen by the Inclusive Research Oversight Board (IROB), which provides strategic governance and ensures coherence across the BRC/ CRF infrastructures in relation to research inclusivity (Fig 1).

The IROB was initially established to embed the principles of IR across the experimental medicine programmes and strategic initiatives of the Manchester BRC/ CRF. It has subsequently expanded to include representatives from the wider Manchester NIHR funded family of programmes (e.g., the Healthtech Research Centre and the Health Determinants Research Collaborative). Therefore, IROB brings together a diverse membership, including patients, citizens, public health professionals, researchers, methodologists, and representatives from partner organisations such as Greater Manchester Integrated Care System, The National Institute for Health and Care Excellence, local authorities and others.

**Fig 2:**
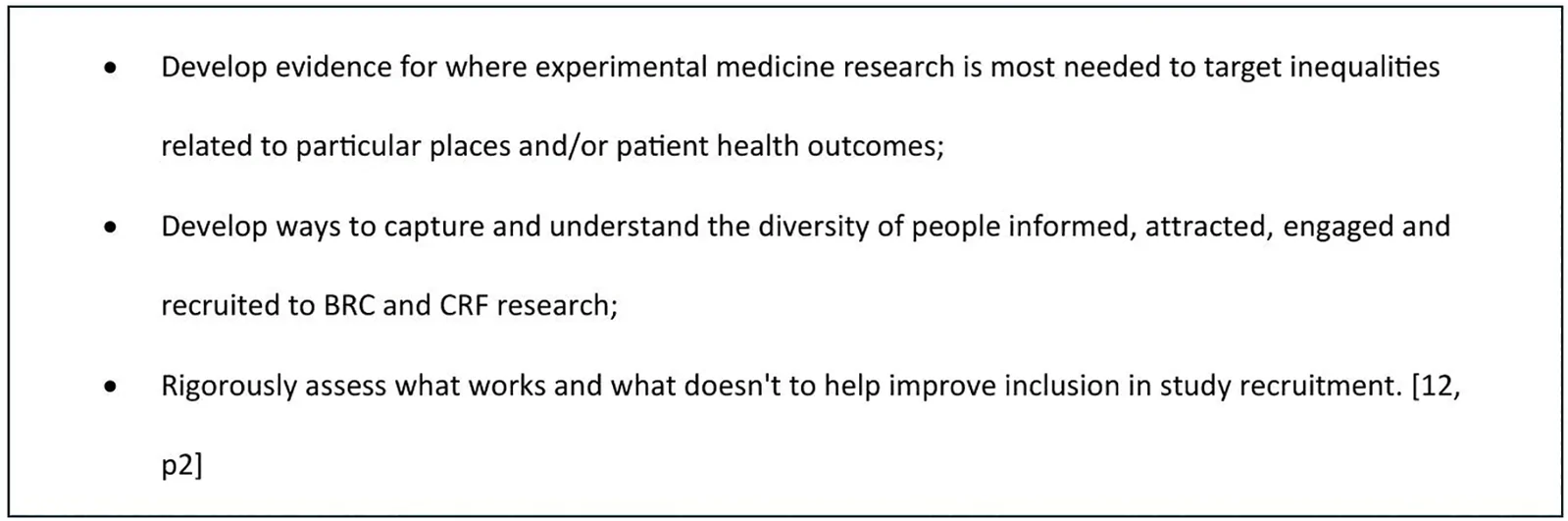
Core objectives of the Inclusive Research Oversight Board (IROB).

Its mission is to provide internal peer review, expert guidance, and methodological support to ensure that inclusivity is integrated across the research cycle – from identifying barriers to inclusion, to understanding impact on research design, and ultimately implementing solutions to maximise the value of inclusive practices. Its core objectives are set out in Fig 2.

Achieving meaningful inclusivity in research requires addressing structural barriers, raising awareness, and expanding participation among under-served groups. The IROB, alongside the EDI, PPIE and IRM teams, work collaboratively to advance health equity and embed inclusive practice across the BRC/ CRF. This includes promoting co-production with patients and the public and fostering diversity within the workforce and student community, to collectively address inclusivity and health equity in the research that Manchester BRC/CRF prioritises, designs, undertakes and disseminates.

#### Complexity and change.

The dynamic and complex nature of the current research landscape necessitates a flexible and adaptive approach to Strategy development, implementation, evaluation and change. It is increasingly recognised that rigid planning cannot account for all contingencies. However, organisations must be capable of responding to external changes to remain competitive and effective [13,14]. In this context the IR Strategy has been designed to be iterative and responsive, able to evolve in line with emerging challenges, opportunities and stakeholder needs. The adaptability is essential for embedding inclusive practices across diverse research environments and ensuring that the Strategy remains relevant and impactful over time.

#### Wider policy context.

In the wider policy context, the National Healthcare Inequalities Improvement Programme [8] includes CORE20PLUS5 [15], a key framework for addressing health inequalities at both local and national levels. These inequalities refer to unfair and avoidable differences in health outcomes across populations and between different groups. This Programme [8] takes CORE20 – the most deprived 20% of the national population, adds PLUS populations and the five clinical areas of focus as shown in Table 2.

**Table 2:** CORE20PLUS5 attributes [adapted from 15].

Core20	PLUS	5
The most deprived 20% of the national population as identified by the national Index of Multiple deprivation (IMD). The IMD has seven domains with indicators relating to a wide range of social determinants of health.	PLUS population groups are identified at a local level. Expected populations could include: ethnic minority communities; people with a learning disability and autistic people; people with multiple long-term health conditions; other groups that share protected characteristics as defined by the Equality Act 2010; and groups experiencing social exclusion called ‘inclusion health groups coastal communities’ (where there may be small areas of high deprivation hidden amongst relative affluence). Other inclusion groups include: people experiencing homelessness, drug and alcohol dependence, vulnerable migrants, Gypsy, Roma and Traveller communities, sex workers, people in contact with the justice system, victims of modern slavery and other socially excluded groups.	Five clinical areas of focus identified as requiring accelerated improvement. Governance for these five focus areas sits with national programmes: • Maternity • Severe mental illness • Chronic respiratory disease • Early cancer diagnosis • Hypertension

The Government’s White Paper on devolution in England [16] contained within it the commitment to furnish Strategic Authorities with “…*a bespoke duty in relation to health improvement and health inequalities.*” This duty will work alongside those that already exist for local governments and their partners, as well as helping to promote a “health in all policies” [17] approach in Strategic Authorities and across their partnerships with the health and care system and others.

#### A research inclusivity framework.

A framework developed by Morrow et al [18], originally applied within the educational sector, identified four key domains within which IR is being developed and implemented, that are equally applicable in IR in health: funding and research (e.g., socially relevant research, IR assessment and equity in research priorities), public and community engagement (e.g., inclusion of diverse communities, intersectional inequalities and greatest need), environments and cultures (e.g., workforce, leadership and career development), and research practices (e.g., under-served groups, recruitment and tools, data, analysis and dissemination).

These domains align and resonate with the BRC/ CRF’s definitions of EDI, PPIE and IRM, the three pillars which underpin the core ideas of Research Inclusivity for Manchester BRC/ CRF:

- *Patient and Public Involvement and Engagement (PPIE):* Ensuring research is shaped by diverse voices and lived experiences, particularly from the under-served and under-represented communities;
- *Equality Diversity and Inclusion (EDI):* Fostering inclusive research environments through workforce diversity, equitable career progression, and diverse leadership;
- *Inclusive Research Methods (IRM):* Advancing the evidence base and implementation of inclusive practices across research funding, design, recruitment, evaluation, analysis and dissemination.

**Fig 3:**
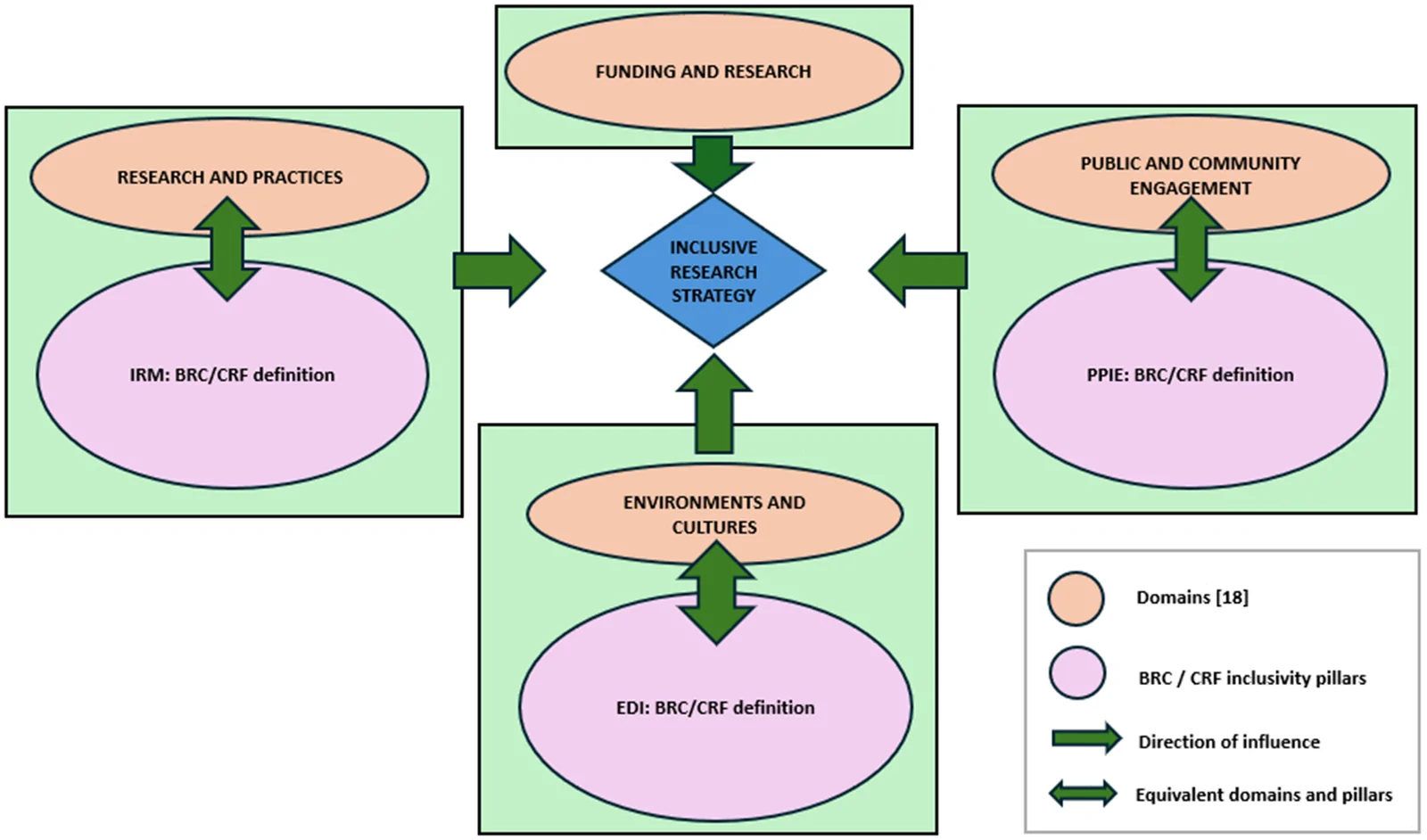
Domains [18] and BRC/CRF inclusivity pillars informing the IR strategy.

Together, these pillars support a more equitable, impactful, and community-responsive research culture. This provides a valuable lens through which the Manchester BRC and CRF can shape, implement and continually refine its IR Strategy. The domains and pillars are illustrated in Fig 3.

#### Developing an Inclusive Research Strategy that reflects diverse perspectives.

Building on the domains [18] and pillars outlined, this section describes the process through which the IR Strategy was developed for the Manchester BRC/ CRF and their partner organisations. The Strategy was designed to complement – rather than replicate – the work of the existing teams focused on PPIE and EDI, both of which have been integral to the BRC since its inception in 2017.

It was also essential that the Strategy aligned with the NIHR’s Research Inclusion Strategy 2022–2027 [19], which defines success through the following principles:

- Embedding equality, diversity, and inclusion across system, culture and processes;
- Building a more diverse research and advisory workforce;
- Expanding access and participation in health and social care research;
- Ensuring visible contributions from all members of the NIHR community;
- Adopting a continuous learning approach to identify what works and what does not across sectors.

The working definition of IR adopted by the Manchester BRC/ CRF was developed proactively, prior to the NIHR’s formal announcements about inclusivity in 2024.

*“Inclusive translational research takes deliberate action to meet the health research needs of different people, to address barriers to inclusion and to promote environments where everyone feels included, respected and valued.”*

This definition was co-created during the first BRC funding cycle (2017–2022) by researchers, the Black Asian & Minority Ethnic Research Advisory Group and Vocal (whose vision is to “bring people and health research together for everyone’s benefit” [20]). Patient and public contributors from across the region played a central role in shaping the BRC and CRF vision and aims and continue to be embedded within the scientific and governance structures of the programmes. The early commitment to IR reflects the leadership of both programmes in advancing research inclusivity locally. The decision to structure inclusivity around the three distinct functions further reinforces this strategic intent.

Subsequent sections of this paper outline the process through which the IR Strategy was co-produced, is being implemented, evaluated and adapted in relation to changing local, regional and national contexts.

## Methods

### Testing knowledge and priorities that existed

In July 2023, a dedicated IRM team was officially established to support the Manchester BRC/ CRF in embedding inclusivity throughout the research cycle. The team’s initial task was to review the three foundational strategies already in place: PPIE [12], EDI [21] and Training and Capacity Building [22]. The objective was to develop a complementary Strategy that would be co-produced by researchers and wider stakeholders, under the governance of the IROB. The Strategy would serve as a framework for implementing, monitoring and advancing inclusive practices across the BRC/ CRF infrastructure. In addition to reviewing strategic documents, the IRM team drew on IROB’s terms of reference and objectives (Fig 2) and early operational insights, including learning from the previous Health Inequalities Steering Group and intelligence gathered from ad hoc conversations with a diverse range of informants from events held across the BRC/ CRF (e.g., PPIE training, Board meetings etc.). This comprehensive approach ensured alignment across existing structures, buy-in and maximised opportunities to embed IR principles across the organisations.

### Ethics

As the work to inform the Strategy was conducted with professionals speaking about their roles (BRC/CRF employees and public contributors were treated as being employed by the BRC/CRF) we used the Manchester University ethics decision tool which confirmed we did not require formal ethics approval for this work (workshop, discussions and ad hoc conversations). For ethical purposes the information gathered was treated as participation and co-production of the Strategy rather than research.

### Workshop

To further inform the Strategy, a half-day workshop was held in September 2023. This event brought together over 60 participants from across the BRC, CRF and partner organisations, representing a wide range of roles – from senior leadership and researchers to our public contributors. Using interactive tools such as Mentimeter [23], attendees were initially invited to share their initial understanding of IR.

### Discovering aims and plans for IR

Workshop participants were then invited to articulate their priorities for IR, both individually and on behalf of their teams. Facilitated by members of the IRM team and colleagues in the University of Manchester’s Public Health department, participants responded to four key prompts:

- What are your short-term aims for IR (years 2–3 of the BRC/ CRF)?
- What are the longer-term aims (4–5 years)?
- How can IROB and the IRM team support your plans for IR?
- How can IROB and the IRM team assist with participatory approaches?

Working in small groups of 5–7 across 10 tables, participants used ‘Well Sorted’ technology [24] to reach consensus and generate four responses to each prompt. To ensure clarity and accountability, participants were encouraged to apply SMART principles – Specific, Measurable, Achievable, Relevant, and Time bound [25] – in formulating their responses.

### Sorting and prioritising aims and plans for IR

Following the initial discussions, participants collaboratively sorted these collective responses into three categories to help the team rank aims:

1. Must be done (high priority);
2. Should be done (medium priority);
3. Could be left out (lower priority).

### One-to-one discussions

Following the workshop, one-to-one discussions were held during October/ November 2023 with three public contributors who either sit on the IROB or are invited to the quarterly specialist IROB meetings. Questions discussed included:

- What are the key elements you’d like to see in the IR Strategy?
- What priorities/ actions are really important to you/ which are less important?
- What is important in terms of IR to the people/ communities you represent?
- What should the overall IR ambitions be – i.e., what should be different by end of this round of the BRC/ CRF funding?

All insights gathered were subject to systematic thematic analysis (STA) [26] by the IRM team, to identify key issues rated to Inclusivity that would form the basis of the Strategy. STA helped to Identify and interpret patterns/themes in data [27]. Braun and Clarke [26] suggest using the six step framework with which the team broadly worked – (1) Familiarisation – carefully reading and reviewing the information gathered; (2) Coding: generation of concise labels for important features; (3) Generating Themes: grouping codes into potential themes; (4) Reviewing Themes: checking the themes back against the information collected; (5) Defining and Naming Themes: Describing the themes; (6) Writing Up: Weaving together the analytic narrative to tell the story. Each of the steps contributes systematically to the development of meaningful insights while allowing the researcher to remain actively engaged with the information. The themes found are described in the following section.

## Findings from the consultations

**Fig 4:**
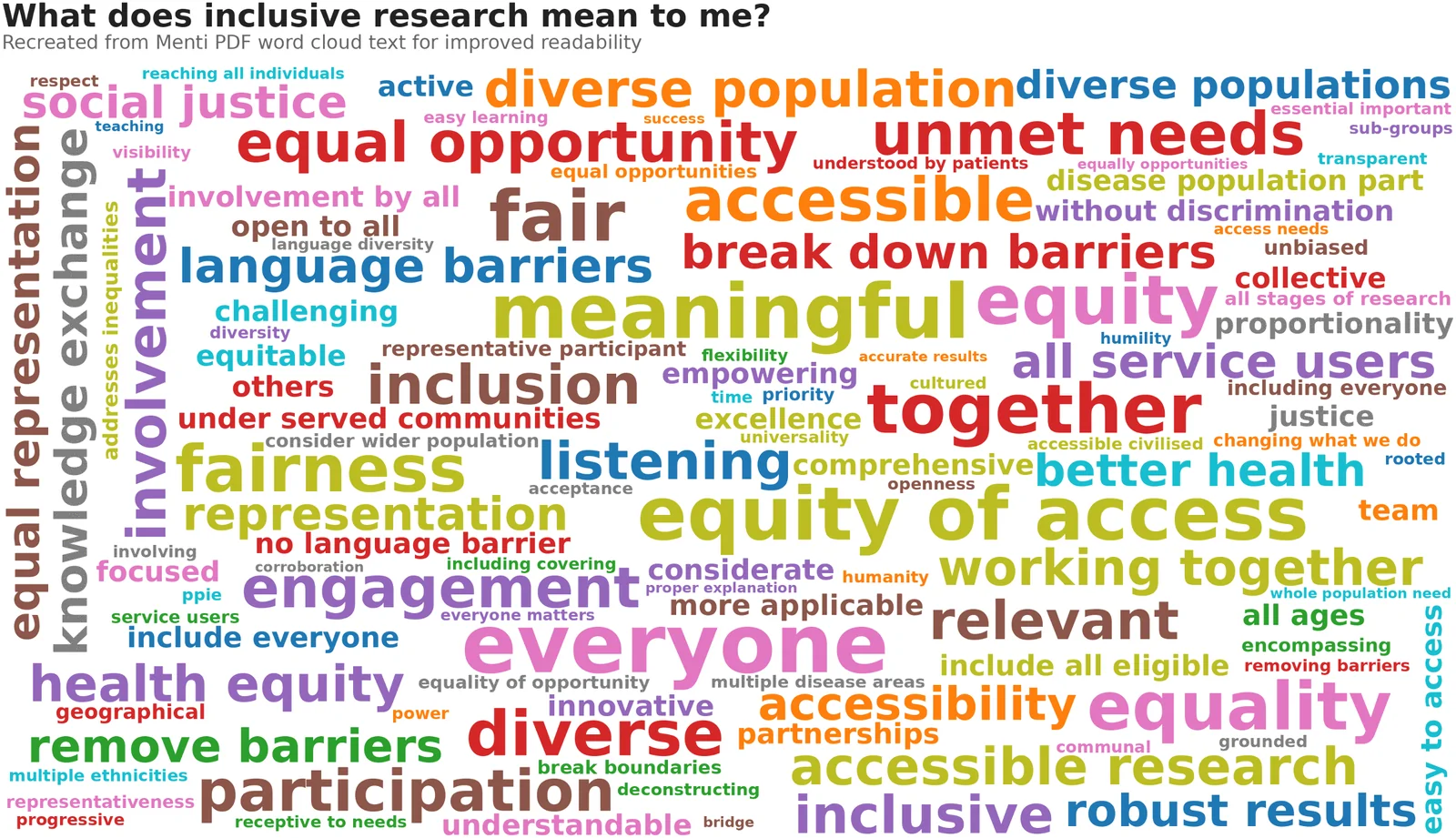
Word cloud.

The workshop revealed varying levels of familiarity with IR across the different parts of the programmes (BRC/ CRF) as shown in the word cloud (Fig 4). This highlighted the need for a shared and consistent understanding of the concept, which would need to be set out in the Strategy.

Using the principles of STA [26] the following recurring themes (named by the IR team) were surfaced by workshop participants as they articulated their priorities for IR, sorting and prioritising their importance:

1. Mapping of inequalities and protected characteristics;
2. Training and capacity building;
3. Working with communities, in wider partnerships and co-production;
4. Resources and practicalities – available resources alongside internal systems and supporting structures within our partner organisations.

Themes were identified as important for development based on various factors including recurring patterns, how often participants mentioned them, or their significance for specific groups (e.g., across BRC/ CRF, different job roles etc.). The themes also related well to Morrow et al’s four key domains [18] as shown in Table 3.

**Table 3:** Comparing the developing IR Strategy themes and Inclusive Research Domains [18].

IR Strategy themes	Domains
Mapping of inequalities and protected characteristics	Inclusive research practices
Training and capacity building	Inclusive research environment and cultures
Working with communities, in wider partnerships and co-production	Inclusive public and community engagement
Resources and practicalities	Inclusive research funding and design

Participants were also asked what support they would find most helpful from the IRM Team and IROB. Key areas of support included: education and training—both general IR awareness and targeted learning around health inequalities; improved communication and visibility of IR, including championing inclusive practices; access to practical resources such as exemplar studies, case studies, checklists, and a centralised resource hub; and ongoing monitoring and evaluation of IR activities (See Supporting Information (S1 File) – Well Sorted [24] exercise outputs).

In addition, respondents shared ideas for future participatory approaches. These included: building a deeper understanding of local populations; increasing awareness of barriers to IR and engagement; identifying high-inequality areas and underserved populations; using relevant data sources to inform inclusive practices; providing accessible support, advice, and resources; and demonstrating the value of IR within experimental medicine research to strengthen its case for wider adoption. This was viewed as important because researchers and delivery teams may be more likely to engage with IR when it is presented not only as an ethical or policy requirement, but as a way to improve study feasibility, recruitment quality, participant experience, external validity and the eventual utility of findings.

In the one-to-one discussions, public contributors emphasised the importance of collaboration across a diverse range of expertise – including medical/ clinical, research, lived experience – working collectively towards a shared goal. They stressed the need to dismantle power dynamics so that all perspectives are genuinely considered and valued. In addition, they called for greater clarity on how the IR Strategy aligns and builds on the existing PPIE and EDI strategies [12,21] within the BRC/ CRF. Crucially public contributors asked for transparency around the Strategy’s intended impact: what it realistically aimed to change, who it is designed for, and how success would be measured – particularly in terms of improving lives at the end of the current five-year BRC/ CRF cycle. Contributors also proposed changes to working practices over the course of this cycle and beyond as summarised in Table 4.

**Table 4:** Proposed changes to working practices.

Change to working practice proposed	Description
**Encouraging honesty and boldness**	Being open to experimentation, sharing what works and what doesn’t, and ensuring feedback is provided to research participants
**Offering researcher training**	Includes insights into diverse communities, the need for integrated health services, and the complexity of interacting health conditions
**Ensuring successful initiatives are scaled thoughtfully to wider populations**	Follow-up to assess effectiveness and an awareness that one-size-fits-all approaches may not be appropriate
**Avoiding repeated engagement with the same communities simply for convenience**	Recognising that over-consultation can lead to narrow representation
**Challenging entrenched attitudes**	Acknowledging that while change is underway, progress remains slow. Contributors noted that IR must move beyond rhetoric to meaningful action.

Discussions with our public contributors also highlighted several key requirements of the developing IR Strategy, which included: making the Strategy simple and accessible to all; incorporating regularly assessed SMART targets [25]; creating short and longer term aims and objectives; building on existing research and evidence; clarifying ownership for all activities and targets; being flexible and responsive as things change and evolve over time (ineffective activities discontinued and successful ones sustained); and strengthening accountability and transparency with honest reflection. These insights are being actively incorporated into the development and implementation of the IR Strategy, shaping its evolution over time.

### Development of the strategy

Taking all the insight described in the previous sections into account within the wider policy context the IR Strategy was developed, closely aligning it with the Manchester BRC/CRF’s existing strategies for PPIE, EDI [12,21], and training and capacity building [22]. It also considers the resources available to the small IRM team over the remainder of the current contract period.

The resulting Strategy is a commitment to building a more inclusive and effective research environment, which will drive forward greater improvements in health and care for patients. This involves taking deliberate action to meet the health research needs of different people, to address barriers to inclusion and to promote environments where everyone feels included, respected and valued. IR seeks to make the invisible, visible and build methodologies throughout the research process, ensuring good science and that research outcomes are relevant and meaningful.

Importantly, the IR Strategy has become a living document—intended to evolve over time. Strategic objectives and associated activities will be reviewed and refreshed regularly through established BRC/CRF governance structures informed by the annual evaluation cycle. The initial themes identified through the consultation process are more fully outlined below. These themes resonate with commonly identified themes found by the analysis of NIHR mandated EDI Strategies [28] of 20 BRCs and 28 CRFs across the country. This research found that for BRCs ‘cultural change in workplaces’ was most commonly identified across objectives for BRCs, with ‘leadership, governance and policy’ for CRFs. Within action plans [28] it was found that both BRCs and CRFs commonly cited ‘research development and delivery’. Manchester’s decision to develop a standalone IR Strategy (with a small supporting team and dedicated resources) to complement its EDI and PPIE Strategies [12,21] allows it to take these broad themes and unpack them further under the five themes set out below.

#### Mapping of inequalities and protected characteristics.

This theme centres on the importance of mapping inequalities and protected characteristics to inform IR practices.

- Participants highlighted the need for data-driven approaches to inclusive recruitment and retention, considering factors such as demographics, geography, culture, and disease prevalence (e.g., through heat maps of disease).
- Discussions focused on identifying relevant data sources, such as baseline figures, dashboards, and patient lists and exploring methodologies for data collection that support IR design and delivery.
- Contributors also identified barriers to broad participation in research, such as restrictive inclusion criteria (e.g., age limits, clinic attendance requirements, or procedures like taking blood), and proposed solutions including translation services, travel reimbursement, and avoiding over-reliance on easily accessible groups (e.g., student populations) for recruitment.

#### Training and capacity building.

This theme reflects participants’ suggestions for strengthening training and development across the BRC and CRF to support IR.

- Contributors expressed a need for greater understanding of IR and the structures within the BRC/CRF that enable it. They proposed a clear and accessible ‘menu of support’ from the IRM team and IROB, which researchers could draw upon throughout their projects.
- The ability to share knowledge and experiences across the BRC, CRF and wider research community was seen as essential. Participants recommended establishing forums to discuss challenges and explore solutions collaboratively.
- There was strong support for the creation of standardised resources, such as case studies and examples of good practice that researchers could refer to when designing and conducting IR.
- Training specifically focused on IR was viewed as a priority, with participants emphasising the importance of being able to measure its impact across the BRC/CRF. Suggestions included regular training events tailored to different parts of the BRC, and the development of ‘Inclusive Research Champions’ to promote and support IR.
- Specific areas where support and training were requested included inclusive grant writing, research methodologies, sampling strategies, quality impact assessments, and approaches to engaging diverse populations.
- Finally, participants highlighted the need for dedicated resources, including funding, to enable meaningful implementation of IR practices.

#### Working with communities, in wider partnerships and co-production.

This theme focused on reducing barriers to participation in experimental medicine research by strengthening relationships with communities and fostering collaborative approaches.

- Participants emphasised the importance of clearly communicating the purpose and benefits of experimental medicine research to targeted populations. Suggestions included improving the visibility and public image of experimental medicine research, building trust with potential participants, and using accessible channels, such as social media, community campaigns, and local champions to share information and encourage engagement.
- In experimental medicine, where studies may be unfamiliar, procedure-heavy or perceived as risky, trusted intermediaries and clear communication were cited as being important for explaining why participation matters, what participation involves, and how research findings will be used.
- Ensuring that research findings are communicated back to participants and communities in meaningful and accessible ways was seen as essential. This could include using infographics, plain language summaries, and working with partners like Vocal [20] to support dissemination.
- Practical ideas for increasing community access to research included taking studies into local settings, such as through mobile research units, and involving community-based researchers, such as religious leaders to facilitate participation.
- The importance of aligning with PPIE structures was highlighted to ensure timely and appropriate engagement with the right people throughout the research process. Relevant co-production methods should be used to support shared decision-making and ownership.
- Participants also stressed the need to demonstrate the value of IR to industrial stakeholders. This could involve engaging them in study site selection, hosting periodic stakeholder events, and showcasing the benefits of inclusive practices for research quality and impact.

#### Resources and practicalities.

This theme captured important practical suggestions that did not fit neatly within the other categories but are essential to the successful implementation of the IR Strategy.

- Participants recommended increasing the visibility and accessibility of the IRM team. Suggestions included introductory meetings with research clusters and themes, drop-in sessions, and regular updates using varied communication formats.
- Several contributors raised concerns about broader research processes that can hinder progress. These included slow or complex systems, multiple layers of sign-off, and financial-related delays, all of which can impact the timely delivery of IR.
- These practical constraints are especially significant for experimental medicine because opportunities to influence inclusion may occur early and within compressed timelines. If IR considerations are introduced only after protocols, budgets, recruitment sites or sponsor requirements have been fixed, the scope for meaningful adaptation can be limited.
- The importance of capturing learning throughout the Strategy’s implementation was emphasised, with a view to using this insight to guide future developments and ensure that IR remains central to the BRC/CRF research agenda.
- Respondents expressed a desire to influence decision-making within research bodies and commercial partners, particularly during funding processes and other key stages of research development.
- CRF participants highlighted challenges around early engagement with research projects, noting a lack of control over study selection and limited availability of Principal Investigators. Barriers included workload pressures, insufficient incentives, and limited protected time for research.

A fifth theme was added by the team to specifically meet the target set by the leadership team and Scientific Advisory Board – that of:

#### Using and creating Inclusive Research Methods.

This theme informed the ambitious target of engaging with researchers and public contributors across the BRC/ CRF programmes to co-produce new methodologies and/ or adapt those in use elsewhere. It reinforced the need for the IRM team to continuously look for research methods to employ for the purpose of understanding the impact of:

- Low inclusivity (in research and trails).
- Inequalities.
- Inequity in healthcare and social care interventions.

This will ultimately help to adapt traditional frameworks/ methods to distribute power and tackle structural inequalities throughout the entire research lifecycle.

### Strategic principles

**Fig 5:**
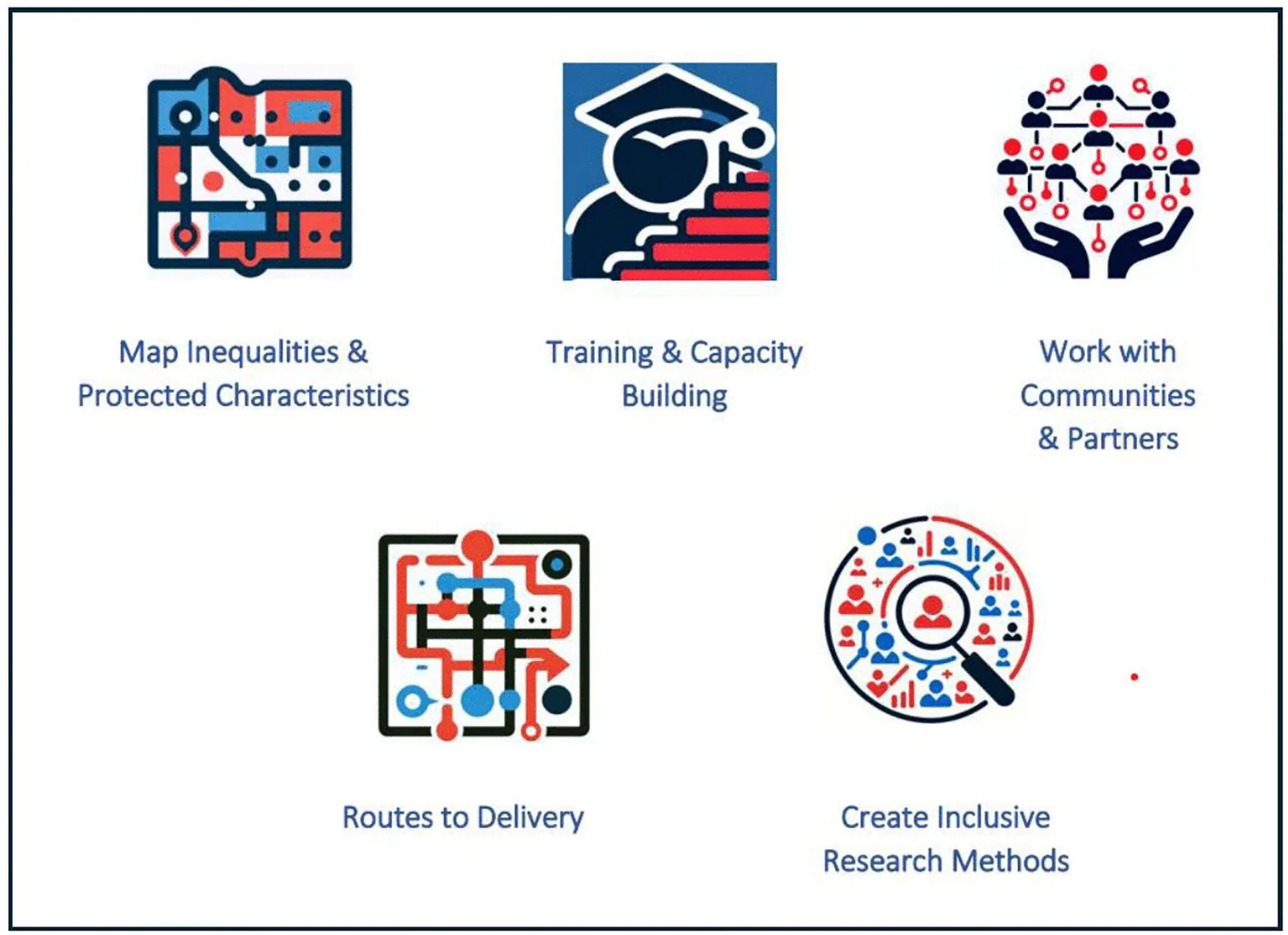
The five strategic principles.

Insights from workshops and wider discussions helped shape the IR Strategy around the five key strategic principles, formed from the themes above: Map inequalities and protected characteristics; Training and capacity building; Work with communities and partners; Routes to delivery and using and creating IRM (Fig 5).

These principles provide the foundation for embedding IR practices across the BRC and CRF. By working in close collaboration with colleagues in EDI, PPIE, and capacity building teams, the IR Strategy aims to foster a cultural shift, one that places proactive inclusion at the heart of research throughout its lifecycle. It also ensures that training and development opportunities related to IR are accessible and relevant to the entire workforce, including students. Importantly, the Strategy brings evidence-led IR into sharper focus across the BRC and CRF infrastructure, enabling meaningful and lasting change for researchers, participants, and the wider community.

For each of the five strategic principles, a series of SMART objectives [25] were developed to guide both short-term and longer-term actions. Progress is being monitored through established governance structures led by the IROB and supported by rapid cycle evaluation methods [29] underpinned by a realist framework [30]. Realist evaluations [30] are often used in implementation science, and they look to understand how and why interventions work, rather than just if an intervention is successful. This is achieved by examining the interplay between identified contexts, mechanisms and outcomes (CMOs) and allows real-time testing of initiatives, generating timely evidence and actionable insights to inform ongoing improvements.

Each objective is supported by a set of proposed activities and initiatives, which will evolve over time based on learning from implementation. These activities will be incorporated into action plans where appropriate, with clear ownership assigned for delivery. Adequate resources will need to be allocated to support the implementation of the Strategy and its objectives, ensuring alignment with governance processes and enabling sustained progress.

### Implementation: strategic principles and aims

**Fig 6:**
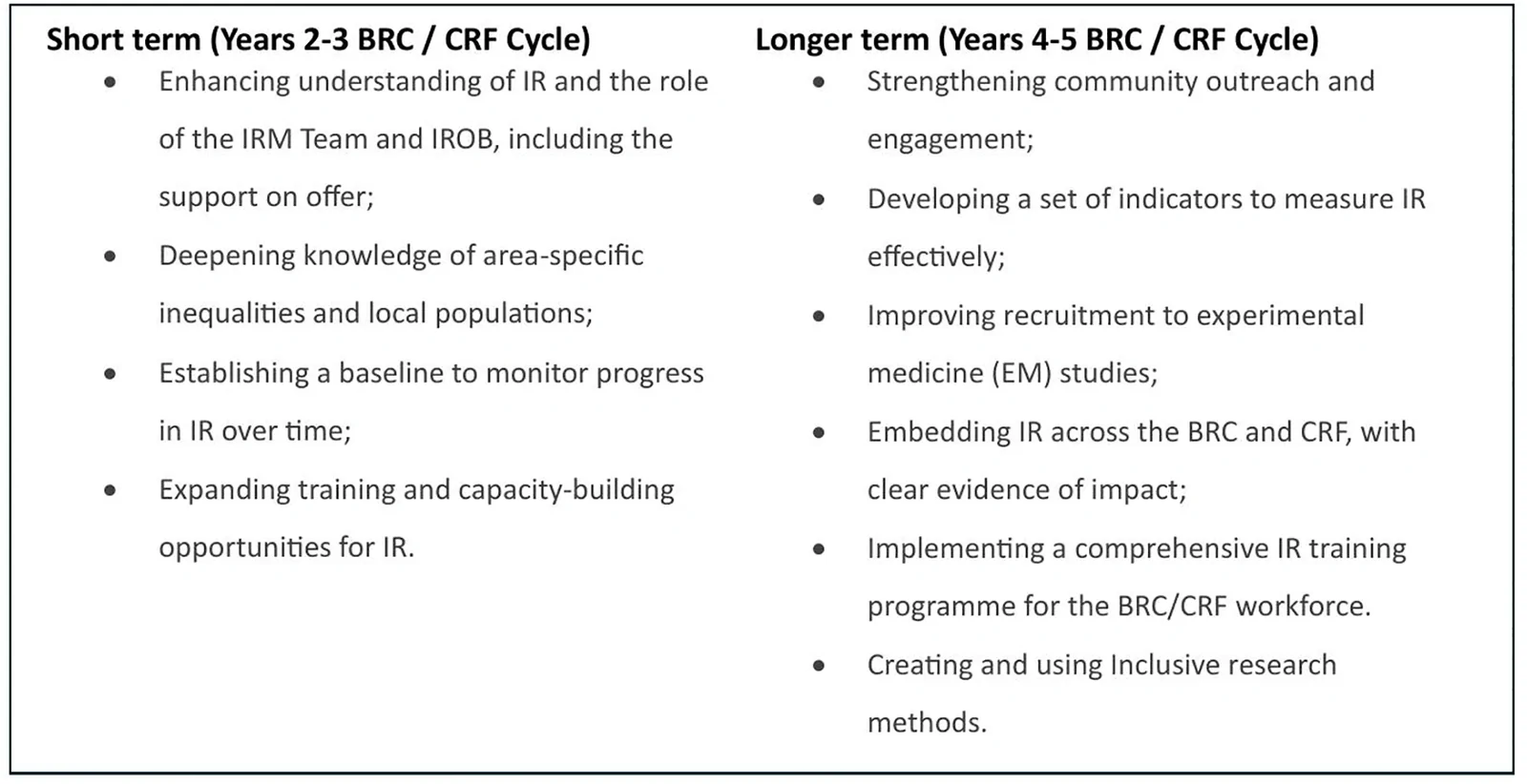
Short and Long term priorities.

Workshop exercises helped identify priority areas for the IR Strategy, as ranked by participants. Each principle was translated into a set of short-term objectives (Years 2–3) and longer term goals (Years 4–5), aligned with the remaining duration of the current BRC/ CRF funding cycle (2024–2027) as shown in Fig 6.

Implementing these principles within experimental medicine requires a balance between ambition and pragmatism. Inclusive approaches must be sufficiently embedded to influence design and delivery, while remaining responsive to protocol requirements, regulatory expectations, participant safety, sponsor timelines and available resources. For this reason, the Strategy does not position IR as a fixed checklist, but as an adaptive process through which teams can identify where inclusivity can be strengthened within the constraints and opportunities (context) of each study.

The overarching aim is to embed a ‘people-in-place’ approach [31] within EM, ensuring that research is both inclusive and responsive to the diverse needs of local populations. This approach is designed to be agile, capable of adapting to emerging challenges and opportunities, while remaining grounded in evidence and community engagement. Central to this Strategy is the principle of team research, defined as: *‘A collaborative effort to address a common goal using the strengths and expertise of a diverse team where contributions of all team members are encouraged, acknowledged, recognised and valued.’* [32].

Team research fosters innovation, reduces error, and enhances relevance and impact of research. It also promotes inclusion, cohesion, and resilience qualities essential for delivering meaningful change across the BRC, CRF and their partner organisations. By working collaboratively across the EDI, PPIE, IRM and capacity building teams, the IR Strategy aims to create a research culture that is inclusive by design and sustainable in practice [33].

### Alignment with existing BRC/ CRF strategies/ visions

This IR Strategy is a critical aspect of our approach but also needed to dovetail and support the other powerful, aligned strategies and visions to drive change across the BRC/ CRF already in place: PPIE [12], EDI [21], and Training and Capacity Building [22]. While each Strategy has a specific focus, together, they form the foundations for a roadmap towards creating a more equal, diverse, and IR environment (Table 5).

**Table 5:** The four complementary visions for BRC/ CRF (IRM, EDI, PPIE and Training and capacity).

Inclusive Research Methods Vision	To create, disseminate and stimulate evidence-based IR principles and research methods in all activities across the BRC and CRF. We will highlight areas of greatest need and inequality to create opportunities for IR to have the highest impact, leaving no-one behind.
**EDI Vision**	To inspire evidence-led EDI practice, supporting a diverse and inclusive workforce across the BRC/ CRF and partner organisations to drive improvements in health and care for all.
**PPIE Vision**	To deliver in close collaboration with Vocal, bringing people and health research together for mutual benefit and has pioneered a joined-up approach to PPIE across GM NIHR infrastructure, ensuring a collaborative community of practice, economies of scale, and tailored approaches. Ultimately, we want to improve the health and lives of people through relevant and inclusive research that includes the voices of everyone, equitably. Through meaningful engagement, we will understand the complex and diverse experience and perspectives of our communities, promoting a more health research confident population.
**Training and Capacity Vision**	To bring together and upskill the BRC community of biomedical researchers, healthcare and hybrid professionals who will contribute to and conduct experimental medicine research. To accelerate and grow a modern experimental medicine workforce, we must reduce fragmentation in the career pathway that negatively impacts less represented groups and contributes to a lack of demographic and experiential diversity.

The IR Strategy also closely aligns to the National level NIHR EDI Strategy [34] and the NIHR IR targets [35].

### Implementation

This section of the paper outlines the ongoing plans to embed IR in our BRC and CRF infrastructures. It is again divided into the five strategic perspectives. Table 6 shows proposals for how each of the five strategic perspectives will be embedded in practice.

**Table 6:** Implementing the five strategic perspectives.

The Five strategic perspectives	Description	Embedding in practice
*Mapping inequalities and protected characteristics*	Using identified patterns of inequality and diversity to help clusters, themes and programmes to identify priorities and improve inclusion in research that the BRC undertakes and disseminates	• collect data, • identify gaps and barriers, • develop action plans, • review progress to improve equity and inclusion for all our workforce, student population and research participants.
*Training and capacity building*	Developing IR training, support and resources to facilitate proactive implementation of IR principles at the earliest stages of research.	• Offer fair and equitable training and support across all levels of the BRC/ CRF • Offer training in various formats (workshops, seminars, self-guided, case stories etc) • Offer training on different platforms (Face to face, online, tool based, website and signposting).
*Working with communities and partners*	Raise the profile of IR across the BRC/ CRF and its external partners and communities within which we work, to embed proactive inclusion across workplace culture and in prioritisation, design and delivery of experimental medicine research.	• Engage systematically with public contributors to co-create and sense check IR initiatives. • Support increased recruitment and greater diversity in research participation. • Facilitate the effective dissemination of research outcomes/results.
*Routes to delivery*	Utilise available resources to create a culture that values and fosters inclusive thinking and collaboration.	• Enable everyone in the BRC/ CRF to apply the lens of inclusivity to their research. • Monitor BRC/ CRF culture and individual projects for inclusivity. • Disseminate our learning widely both within and external to the BRC/ CRF. • Establish a positive legacy of impactful change for IR.
*Using and creating Inclusive Research Methods*	Support those undertaking research to better understand and overcome issues of low inclusivity (trial and research projects), inequalities of health and care outcomes, and inequity in health and care interventions.	• Engage with methodologists, researchers and public contributors across the BRC/ CRF programmes to co-produce new methodologies and/ or adapt those in use elsewhere.

Successful implementation of this Strategy, alongside those for PPIE and EDI [12,21] for the BRC/ CRF, and training and capacity-building for the BRC [22], should drive significant progress towards creating an IR culture across Manchester BRC, CRF and partners’ infrastructures that values proactive inclusion, fosters diversity and promotes equity across its workforce and within research.

#### Monitoring.

To assess progress and ensure the IR Strategy remains responsive and impactful, Rapid Cycle Evaluation is being conducted [29]. These evaluations are designed to capture stakeholder perspectives on how messages and practices related to research inclusivity are being received, understood and enacted across the BRC/ CRF programmes.

The first evaluation (baseline) took place in autumn 2024, with the second in Spring 2025 (currently being analysed). This follow-up revisited recommendations from the initial round, assesses current levels of understanding around IR, identifies emerging opportunities, and explores ongoing barriers to implementation. It will be used to generate a new set of actionable recommendations for the remainder of this BRC/ CRF Cycle. This monitoring activity is set within the broader context of national developments in research inclusivity, including evolving funder requirements and expectations. The insights gained will help ensure the Strategy remains aligned with both local priorities and national policy, supporting continuous improvement and meaningful change.

#### Adapting the strategy.

The results and recommendations from the first Rapid Cycle Evaluation (RCE) conducted in Autumn 2024 (conducted prior to the publication of the IR Strategy) showed that by examining the BRC’s components, processes and outputs related to IR, insights could be gained into what was working and what was not at the early stage of implementation.

Overall, respondents recognised that the BRC’s commitment to IR was beginning to gain some momentum. They were positive about the development of the IR Strategy as it included substantial stakeholder engagement, informed by the BRC context specifically, and had not just applied national guidelines and frameworks. However, although there was general support for the principle of IR as a concept, the need for more clarity around its constituent parts, to allow greater understanding and integration of inclusive practices into the everyday activity of the BRC was raised.

Having adequate resources (personnel and budget) to support Inclusive Research, from core support (IROB and its components, EDI, IRM and PPIE teams) and within the BRC Clusters and Themes, was highlighted as important. The development of clear internal and external communication mechanisms and resources (videos, case stories, good practice) should aid this. The research concluded that IR practices need to be supported by all in the BRC, from the leadership team to researchers and PhD students, to aid buy-in. This will help make research carried out within the BRC more representative of the populations that it serves and the results more applicable to a wider diversity of communities. For this to be monitored, effective data needs to be continuously collected in a standardised form, across research projects, to enable progress to be identified over time.

Following the initial RCE, the NIHR also published new guidance requiring IR design as a formal condition of future funding [2]. Combined this prompted a re-evaluation of the IR Strategy to ensure alignment with the emerging findings from the RCE and the ten steps outlined by NIHR for embedding inclusion into research and grant applications. Each step is accompanied by key considerations for implementation within the BRC/ CRF context, as shown in Table 7.

**Table 7:** NIHR 10 steps and associated considerations (adapted from [2]).

Step	Step description	Considerations
1	Substantiate your research inclusion statements	Move beyond good intentions—ensure the research team has the skills and experience to deliver Inclusive Research.
2	Ensure you know which inequalities are relevant to your field	Conduct literature reviews and other scoping activities to uncover hidden or under-recognised inequalities.
3	Include under-served areas in research	Identify regions with high need but low research activity; consider alternatives beyond traditional locations and groups.
4	Justify your research sample	Review exclusion criteria, collect EDI data, and consider factors such as age, language, and accessibility.
5	Specify which demographic data you will collect and how you plan to use it?	Clarify data collection methods and how demographic insights will inform research design and analysis.
6	Budget for inclusion	Transparently outline costs associated with inclusive practices and demonstrate value for money.
7	Consider how methodological innovation could overcome exclusionary aspects of conventional methods	Explore participatory approaches, community-based recruitment, and alternatives to traditional methodologies.
8	Demonstrate how research can be shaped by diverse and inclusive Patient and Public Involvement and Engagement (PPIE)	Ensure diversity in PPIE contributors, address barriers to involvement (e.g., geography, socio-economic status, language), and centre lived experience.
9	Plan inclusive and impactful approaches to knowledge mobilisation	Develop knowledge mobilisation plans with diverse audiences in mind, identifying key messages and dissemination routes.
10	Use resources available	Leverage existing tools and guidance, such as the Research Support Service EDI toolkit [36], to support Inclusive Research planning and delivery.

Referring to this NIHR framework (10 steps [2]) has ensured that the IR Strategy remains responsive to national policy developments and supports researchers in meeting funder expectations. It also reinforces the commitment to embedding inclusion throughout the research lifecycle, from design and recruitment to dissemination and impact.

## Reflections on the process and conclusion

The development of a published IR Strategy for the Manchester BRC and CRF [37] has been a challenging, lengthy, complex and iterative process, shaped by the diverse perspectives of stakeholders, local priorities, the policy context and latterly evolving national guidance. Aligning this new Strategy with existing frameworks at both local [12,21,22] and national levels [19], building IR cultures has required careful co-ordination and responsiveness to the dynamic context. The process has highlighted the importance of inclusive governance, transparent co-production and strategic adaptability. Strategy implementation is influenced by human dynamics, which can both enable and challenge progress [38]. Recognising this, the IR Strategy has been designed to be flexible, evidence-informed, and grounded in the needs of diverse stakeholders.

The main strengths of the process undertaken have included:

- Strong co-production and engagement with stakeholders – the Strategy was co-produced with a wide range of partners with multiple engagement methods which enhance the legitimacy, relevance and buy-in of the Strategy. Public contributors played a meaningful role, including changing power dynamics and shaping priorities.
- Clear alignment with existing internal, policy and funder priorities – the Strategy dovetails and enhances the existing Manchester NRC/ CRF strategies [12,21,22] is aligned with NIHR shifts (IR as a condition of funding, the NIHR 10 steps to inclusion and the NIHR inclusion Strategy). It considers national frameworks such as Core20PLUS5 [15], which strengthens policy relevance and transferability.
- Integration with existing organisational structures – rather than duplicating existing EDI, PPIE and Training and Capacity proposals, the strategy is positioned as complementary and governance through the IROB provides clarity, accountability and system-level coherence.
- Conceptually robust and theoretically informed – the Strategy is grounded in theoretical and analytical frameworks, e.g., Morrow et als Domains [18], SMART objectives [25], rapid cycle evaluation [29] and there is a recognition of practical challenges such as resources, systems and practical barriers.
- Attention to cultural and system change – the Strategy encourages thought around research culture, workforce capability, governance and methods, and emphasises a team research approach [32] and promotion of inclusivity as ‘business as usual’ thus promoting sustainability.

The benefits and challenges of IR are particularly visible in experimental medicine. On the one hand, inclusive approaches can improve the relevance, acceptability and potential generalisability of research by ensuring that study questions, recruitment strategies, participant materials, procedures and dissemination plans are informed by the populations most affected by the conditions under investigation. This may strengthen recruitment, retention, trust and the eventual applicability of findings. Conversely, experimental medicine studies often involve complex protocols, specialist facilities, intensive participant commitments, safety considerations, regulatory requirements, and sponsor-led timelines. These features can make inclusion more difficult to operationalise, particularly where protocols are predetermined or where delivery teams have limited influence over study design. The Manchester BRC/CRF IR Strategy therefore seeks to address inclusion as both a methodological and organisational challenge, recommending early planning, adequate resourcing, shared accountability and continuous learning.

The main limitations identified included:

- Early stage evidence of impact – to date evidence is largely process-focussed rather than outcome focussed, and demonstratable impacts on recruitment, representativeness, research quality and healthcare outcomes, though anticipated, are not yet experienced.
- Resource dependency and scalability – successful implementation of the Strategy relies on sustained investment (time, funding, specialist expertise) which could limit scalability or replication in less well-resourced and supported contexts. The small size of the IRM team may also constrain pace and reach across the large, complex programmes.
- Variability in understanding and readiness – the initial workshop showed uneven familiarity with IR suggesting embedding the Strategy consistently across the different elements of large programmes could be challenging. Cultural change may also be slower in parts of the programme where IR is perceived as an additional burden rather than integral to good science.
- Buy-in and action by Senior Leadership **–** effective senior leadership commitment must be demonstrated not only through verbal endorsement, such as support for the IR Strategy, but also through tangible action and delivery, ensuring that all programme members observe a consistent and organisation-wide effort to embed research inclusivity at every level.
- One Strategy for Multiple Programmes – as a joint Strategy for the Manchester BRC and CRF, programme-specific needs are addressed through consultation but at times challenges within the CRF can be obscured; for example, protocols are often predetermined by sponsors before reaching the CRF, limiting influence over study design, while recruitment targets may prioritise studies that are easier to enrol, reflecting the practical tensions CRFs must manage between sponsor expectations and the goal of IR.
- Ambition vs pragmatism – the Strategy is intentionally ambitious (e.g., inclusion of methodological innovation, system-wide change) but trade-offs between ideal inclusivity and operational constraints exist. Researchers/ clinicians working under time, funding and regulatory pressures may struggle to implement all elements equally.
- Generalisability beyond the Manchester context – the Strategy is context-specific, rooted in Manchester’s BRC/ CRF infrastructure, partnerships and populations. While the development processes of the Strategy are replicable, adaptation would be necessary in other local circumstances.
- Measurement challenges – although the Strategy commits to developing indicators and evaluation, measuring inclusivity meaningfully remains complex, and risks remain around over-reliance on proxy measures or data that may not fully capture lived experience or intersectionality.

The limitations identified reflect the inherent complexity of delivering IR at scale across diverse programmes, and highlight the need for ongoing learning, evaluation, and adaptation.

Beyond its relevance to the Manchester BRC/CRF context, this work contributes to an emerging evidence base on how IR can be embedded within large translational research infrastructures. The Strategy demonstrates that inclusivity can be approached not simply as a policy requirement or compliance activity, but as a strategic, methodological and cultural endeavour supported by dedicated governance, specialist expertise, co-production and ongoing evaluation. For other BRCs, CRFs and research organisations responding to increasing expectations from funders, regulators and communities, the approach described here offers a practical framework that can be adapted to local circumstances. In particular, the integration of EDI, PPIE and IRM through a shared governance/ support structure, alongside the explicit focus on developing and evaluating IR methods, provides one potential model for translating national ambitions for IR into organisational practice.

The decision to disaggregate research inclusion into its core components – EDI, IRM and PPIE, was both ambitious and forward-looking, particularly in advance of widespread funder requirements. While developing a cohesive Strategy to support this approach has been challenging, early indications suggest that IR principles are becoming embedded within the Manchester BRC/CRF culture. Looking ahead, successful implementation will rely on sustained stakeholder engagement, robust monitoring mechanisms, and a continued commitment to iterative learning and improvement. The participatory and adaptive processes underpinning this work enhance its relevance, legitimacy, and institutional ownership. In this context, annual Rapid Cycle Evaluations [29] will play a critical role in assessing progress, informing refinements, and ensuring accountability. While developed within the Manchester context, the approach described demonstrates how dedicated leadership, governance and methodological innovation can help research organisations translate the principles of IR into sustainable organisational change. In experimental medicine, this is particularly important because the populations included in early translational research can shape the evidence, innovations and care pathways that follow. Ultimately, the Strategy seeks to cultivate a research culture that is inclusive by design, one that values diversity, prioritises equity, and delivers research that is meaningful and impactful for all.

## Supporting information

S1 File Well Sorted exercise outputs. (PDF)

## References

[1] Hunt L, Nielsen MW, Schiebinger L. A framework for sex, gender, and diversity analysis in research. Science. 2022;377(6614):1492–5. doi: 10.1126/science.abp9775 36173857

[2] National Institute for Health and Care Research. Inclusive research design to become an NIHR condition of funding. 2024. https://www.nihr.ac.uk/inclusive-research-design-become-nihr-condition-funding

[3] Child SA, Mulligan C, Pavlov I, Bryan S, Li L, Knowles RL. Developing the evidence-base to inform policy on inclusive research design. R Soc Open Sci. 2025;12(3):241380. doi: 10.1098/rsos.241380 40109939 PMC11919494

[4] Asmal L, Lamp G, Tan EJ. Considerations for improving diversity, equity and inclusivity within research designs and teams. Psychiatry Res. 2022;307:114295. doi: 10.1016/j.psychres.2021.114295 34871875

[5] Moorley C, Northway R, Oshikanlu R. Diversity and inclusion in research. In: Jackson D, Halcomb E, Walthall H, editors. Navigating the maze of research: Enhancing nursing and midwifery practice. 6th ed. Elsevier. 2023:154–66.

[6] Yu J, Elliott M, Curtis M, Pontin D, Wallace S, Wallace C. Promoting Inclusivity in Research: Lessons From Four Group Concept Mapping Studies. International Journal of QualitativeMethods. 2025;24. doi: 10.1177/16094069251329732

[7] National Institute for Health and Care Research NIHR. Best research for best health: The next chapter. 2021. https://www.nihr.ac.uk/documents/best-research-for-best-health-the-next-chapter/27778

[8] National Healthcare Inequalities Improvement Programme. 2021. https://www.england.nhs.uk/about/equality/equality-hub/national-healthcare-inequalities-improvement-programme/

[9] Nind M. The practical wisdom of inclusive research. Qualitative Research. 2017;17(3):278–88. doi: 10.1177/1468794117708123

[10] Walmsley J, Johnson K. Inclusive research with people with learning disabilities: past, present and futures. London: Jessica Kingsley. 2003.

[11] Barton A. Embed, Build, Accelerate – Manchester BRC Director’s Blog – February 2025. 2025. https://www.manchesterbrc.nihr.ac.uk/news-and-events/embed-build-accelerate-manchester-brc-directors-blog-february-2025/

[12] NIHR Manchester Biomedical Research Centre and Clinical Research Facility. Public and Patient Involvement, Engagement and Participation (PPIE) Strategy 2022-2027: Bringing people and health research together for everyone’s benefit. 2022. https://www.manchesterbrc.nihr.ac.uk/wp-content/uploads/2023/05/NIHR-Manchester-BRC-CRF-PPIEP-strategy.pdf

[13] Sudarsanam S, Lai J. Corporate financial distress and turnaround strategies: an empirical analysis. Br J Manag. 2001;12(3):183–99. doi: 10.1111/1467-8551.00193

[14] Holm CG, Kringelum L, Anand A. Creating effective strategy implementation: a systematic review of managerial and organizational levers. Rev Manag Sci. 2025. doi: 10.1007/s11846-025-00880-3

[15] NHS England. Core20plus5. 2021. https://www.england.nhs.uk/about/equality/equality-hub/national-healthcare-inequalities-improvement-programme/core20plus5/

[16] Ministry of Housing, Communities and Local Government. English Devolution White Paper: Policy Paper. 2024. https://www.gov.uk/government/publications/english-devolution-white-paper-power-and-partnership-foundations-for-growth/english-devolution-white-paper

[17] WHO. Health in all policies: framework for country action. 2014. https://www.afro.who.int/sites/default/files/2017-06/140120HPRHiAPFramework.pdf 10.1093/heapro/dau035 25217354

[18] Morrow E, Vandrevala T, Ross F. Building inclusive research cultures– how can we rise above EDI cynicism? Health Education Policy Institute Blog. 2025. https://www.hepi.ac.uk/2025/03/05/building-inclusive-research-cultures-how-can-we-rise-above-edi-cynicism/

[19] National Institute for Health and Care Research NIHR. Research Inclusion Strategy 2022-2027. 2022. https://www.nihr.ac.uk/about-us/who-we-are/research-inclusion/strategy-2022-27

[20] Vocal. 2023. https://wearevocal.org/who-we-are

[21] NIHR Manchester Biomedical Research Centre and Clinical Research Facility. Equality, Diversity & Inclusion Strategy 2022-2027. NIHR Manchester Biomedical Research Centre and Clinical Research Facility. 2022. https://www.manchesterbrc.nihr.ac.uk/about-us/inclusive-research/equality-diversity-and-inclusion-strategy-2022-27/

[22] NIHR Manchester Biomedical Research Centre. Training and Capacity Strategy 2022-2027. 2022.

[23] Mentimeter Technology. https://www.mentimeter.com

[24] Well Sorted Technology. https://www.well-sorted.org

[25] Doran GT. There’s a SMART way to write management’s goals and objectives. Management Review. 1981;70(11):35–6.

[26] Braun V, Clarke V. Using thematic analysis in psychology. Qualitative Research in Psychology. 2006;3(2):77–101. doi: 10.1191/1478088706qp063oa

[27] Ahmed SK, Mohammed RA, Nashwan AJ, Ibrahim RH, Abdalla AQ, M. Ameen BM, et al. Using thematic analysis in qualitative research. Journal of Medicine, Surgery, and Public Health. 2025;6:100198. doi: 10.1016/j.glmedi.2025.100198

[28] Hua P, Dawson S, Phillips H. Equality, diversity and inclusion strategies of NIHR biomedical research centres and clinical research facilities across England: a qualitative content analysis. BMJ Open. 2026;16(2):e109321. doi: 10.1136/bmjopen-2025-109321 41713919 PMC12927318

[29] Chisnall G, Kumpunen S, Vindrola-Padros C. Rapid qualitative research. In: Tierney RobertJ, Rizvi Fazal, Ercikan Kadriye, editors. International Encyclopaedia of Education. Fourth ed. Elsevier. 2023:323–35. doi: 10.1016/B978-0-12-818630-5.11083-8

[30] Pawson R, Tilley N. Realistic Evaluation. Sage. 1997.

[31] Towers R. Understanding people in places. 2024. https://publicpolicydesign.blog.gov.uk/2024/03/15/understanding-people-in-places/

[32] Pankhurst Institute. Team research. 2023. https://www.teamresearch.manchester.ac.uk/glossary/

[33] Salas E, Linhardt R, Fernández Castillo G. The Science (and Practice) of Teamwork: A Commentary on Forty Years of Progress. Small Group Research. 2024;56(3):392–425. doi: 10.1177/10464964241274119

[34] National Institute for Health and Care Research (NIHR). Equality, Diversity and Inclusion Strategy 2022-2027. 2022. https://www.nihr.ac.uk/documents/equality-diversity-and-inclusion-strategy-2022-2027/31295

[35] National Institute for Health and Care Research NIHR. Why we need more inclusive research? 2021. https://evidence.nihr.ac.uk/collection/why-we-need-more-inclusive-research/

[36] Research Support Service. EDI toolkit. https://www.rssleicesterresources.org.uk/edi-toolkit. 2022, updated 2024.

[37] NIHR Manchester Biomedical Research Centre and Clinical Research Facility. Inclusive Research Strategy 2022-2027. 2025. https://www.manchesterbrc.nihr.ac.uk/about-us/inclusive-research/

[38] Lê JK, Jarzabkowski PA. The role of task and process conflict in strategizing. Br J Manag. 2015;26(3):439–62. doi: 10.1111/1467-8551.12076

