# Supplementary material for: Developing an inclusive research (methods) strategy for the Manchester Biomedical Research Centre and Clinical Research Facility
Source: PLoS One. 2026 Sep 16;21(9):e0357608. doi: 10.1371/journal.pone.0357608 (PMC13581002; doi:10.1371/journal.pone.0357608)
Supplement: Supplementary file 1 [file pone.0357608.s001.pdf]

Supporting Information (S1)

Well Sorted Exercise Outputs

Dendrogram, tree map and heat map created from answers provided by respondents at the workshop, within the Well Sorted [24] technology

Types of visuals used

A dendrogram is a graphical representation used to depict the hierarchical relationships among a set of objects, typically generated as the output of hierarchical clustering procedures. It provides a structured visualisation in which observations are successively merged or divided based on similarity measures. A primary analytical purpose of the dendrogram is to inform decisions regarding the optimal partitioning of data into distinct clusters. Interpretation relies principally on the vertical position (height) at which branches join, as this reflects the relative dissimilarity between the grouped objects.

A treemap is a space-filling visualisation technique consisting of a series of nested rectangles that represent hierarchical categories within a dataset. Each rectangle corresponds to a subcategory within a broader classification, enabling the representation of complex hierarchical structures within a constrained visual space. Treemaps facilitate rapid comparison of both relative magnitudes and structural relationships. In this application, colour encoding is used to denote categorical priorities, specifically distinguishing between “must be done” (red), “should be done” (blue), and “would be nice to be done” (green), thereby enhancing interpretability.

A heatmap is a data visualisation method in which values are encoded as colours within a matrix or grid structure, enabling the rapid identification of patterns, trends, and outliers. By mapping numerical values to a colour gradient, typically ranging from cooler tones (e.g., blue) to warmer tones (e.g., red), heatmaps provide an intuitive overview of variation across two dimensions. Higher-intensity colours correspond to greater magnitudes or activity levels, while lower-intensity colours indicate reduced values, supporting efficient visual comparison across the dataset.

Q1: What are your short term aims (0-2 years) for inclusive research?

Dendrogram, tree map and heat map created from answers provided by respondents at the workshop

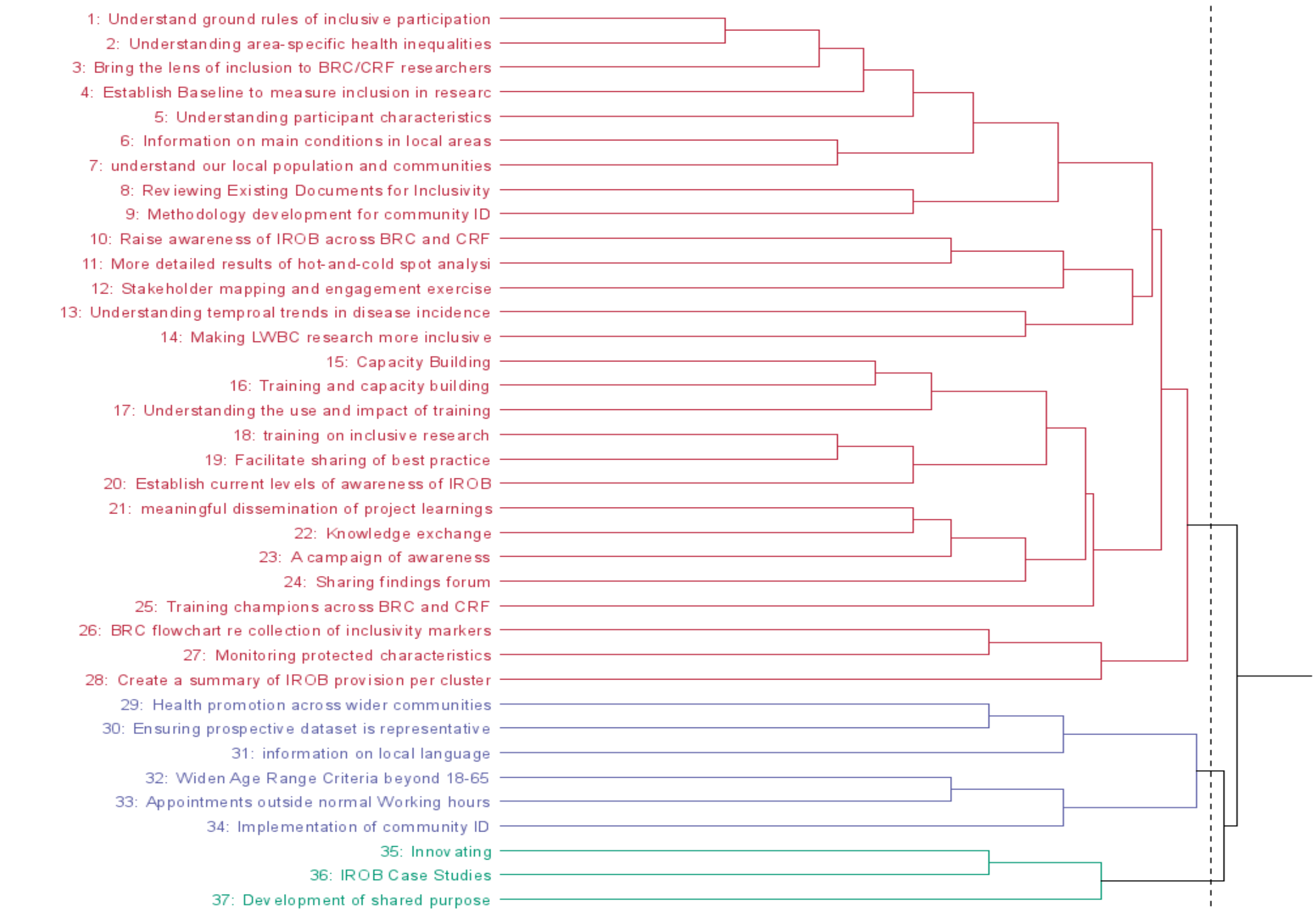

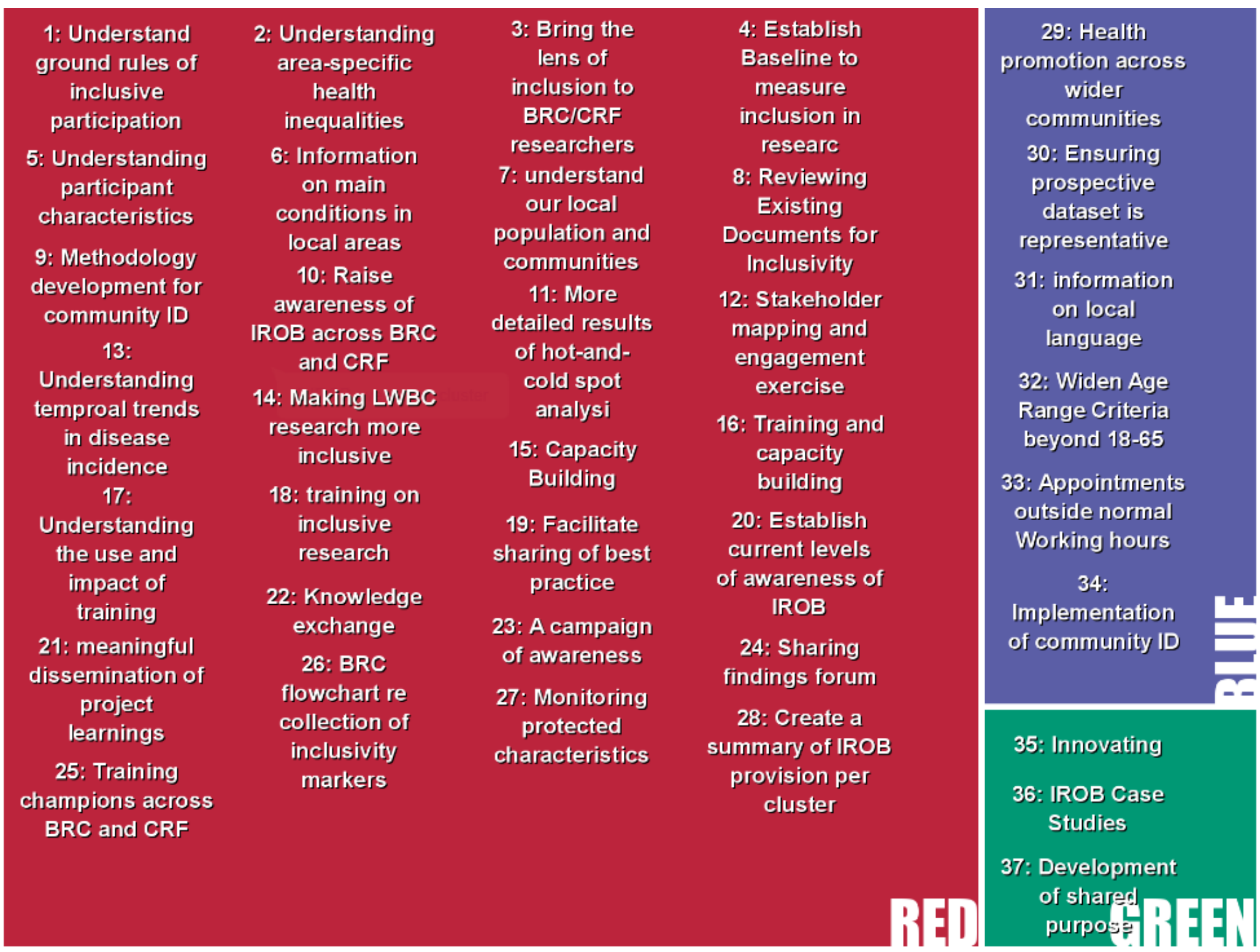

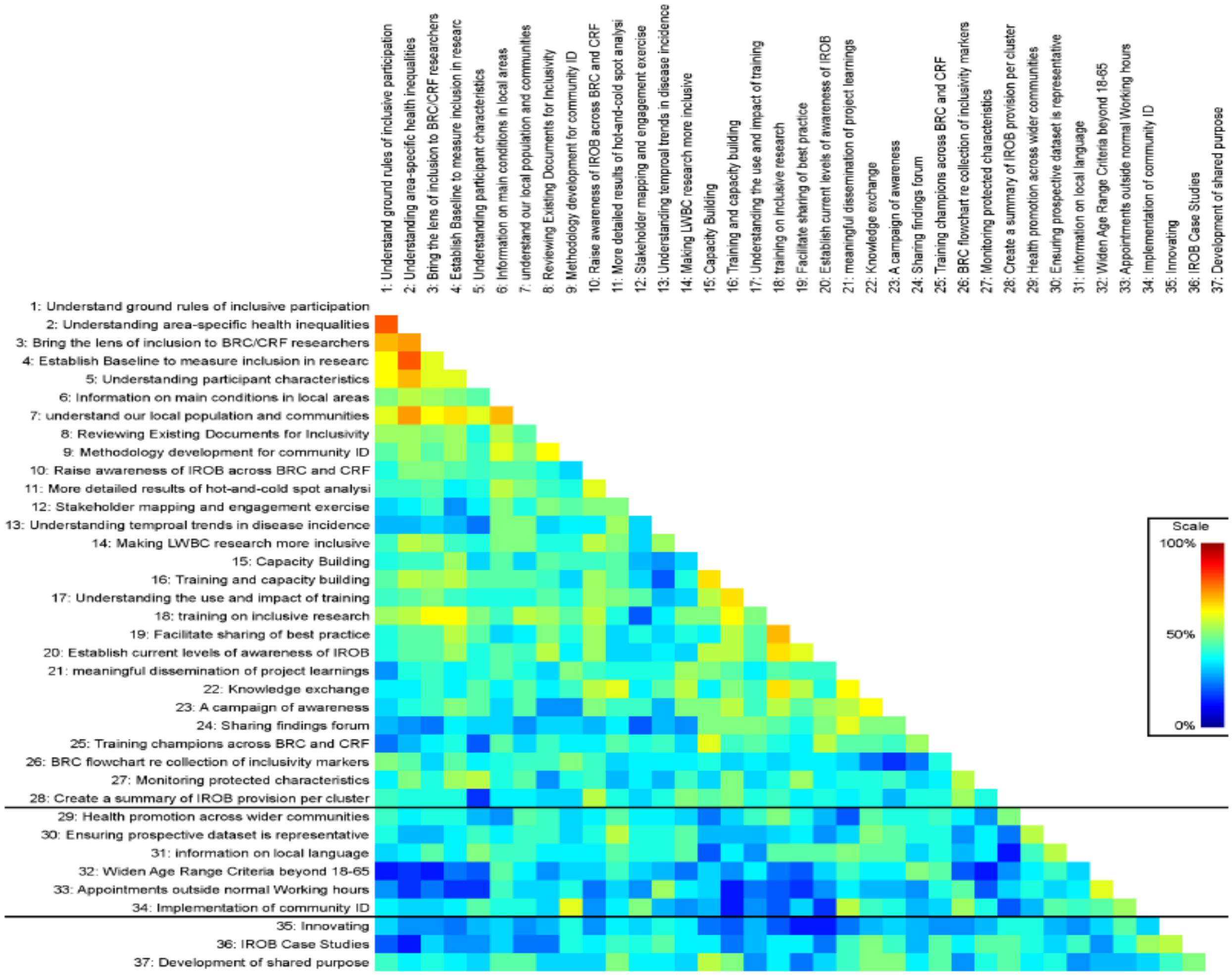
1: Understand ground rules of inclusive participation
2: Understanding area-specific health inequalities
3: Bring the lens of inclusion to BRC/CRF researchers
4: Establish Baseline to measure inclusion in researc
5: Understanding participant characteristics
6: Information on main conditions in local areas
7: understand our local population and communities
8: Reviewing Existing Documents for Inclusivity
9: Methodology development for community ID
10: Raise awareness of IROB across BRC and CRF
11: More detailed results of hot-and-cold spot analysi
12: Stakeholder mapping and engagement exercise
13: Understanding temproal trends in disease incidence
14: Making LWBC research more inclusive
15: Capacity Building
16: Training and capacity building
17: Understanding the use and impact of training
18: training on inclusive research
19: Facilitate sharing of best practice
20: Establish current levels of awareness of IROB
21: meaningful dissemination of project learnings
22: Knowledge exchange
23: A campaign of awareness
24: Sharing findings forum
25: Training champions across BRC and CRF
26: BRC flowchart re collection of inclusivity markers
27: Monitoring protected characteristics
28: Create a summary of IROB provision per cluster
29: Health promotion across wider communities
30: Ensuring prospective dataset is representative
31: information on local language
32: Widen Age Range Criteria beyond 18-65
33: Appointments outside normal Working hours
34: Implementation of community ID
35: Innovating
36: IROB Case Studies
37: Development of shared purpose
Scale
100%
50%
0%

Q2: What are your long term aims (2-4 years) for inclusive research?

Dendrogram, tree map and heat map created from answers provided by respondents at the workshop

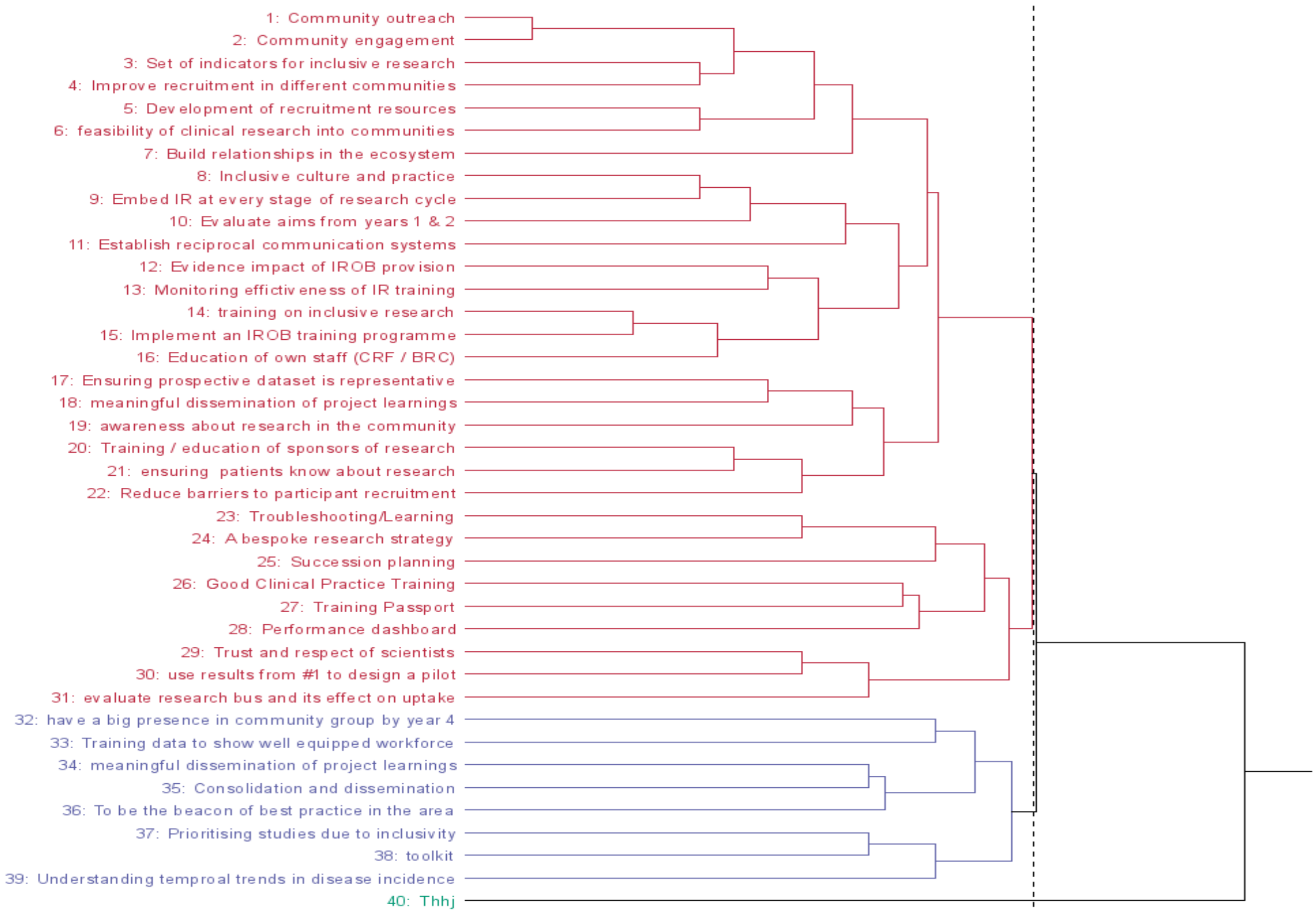

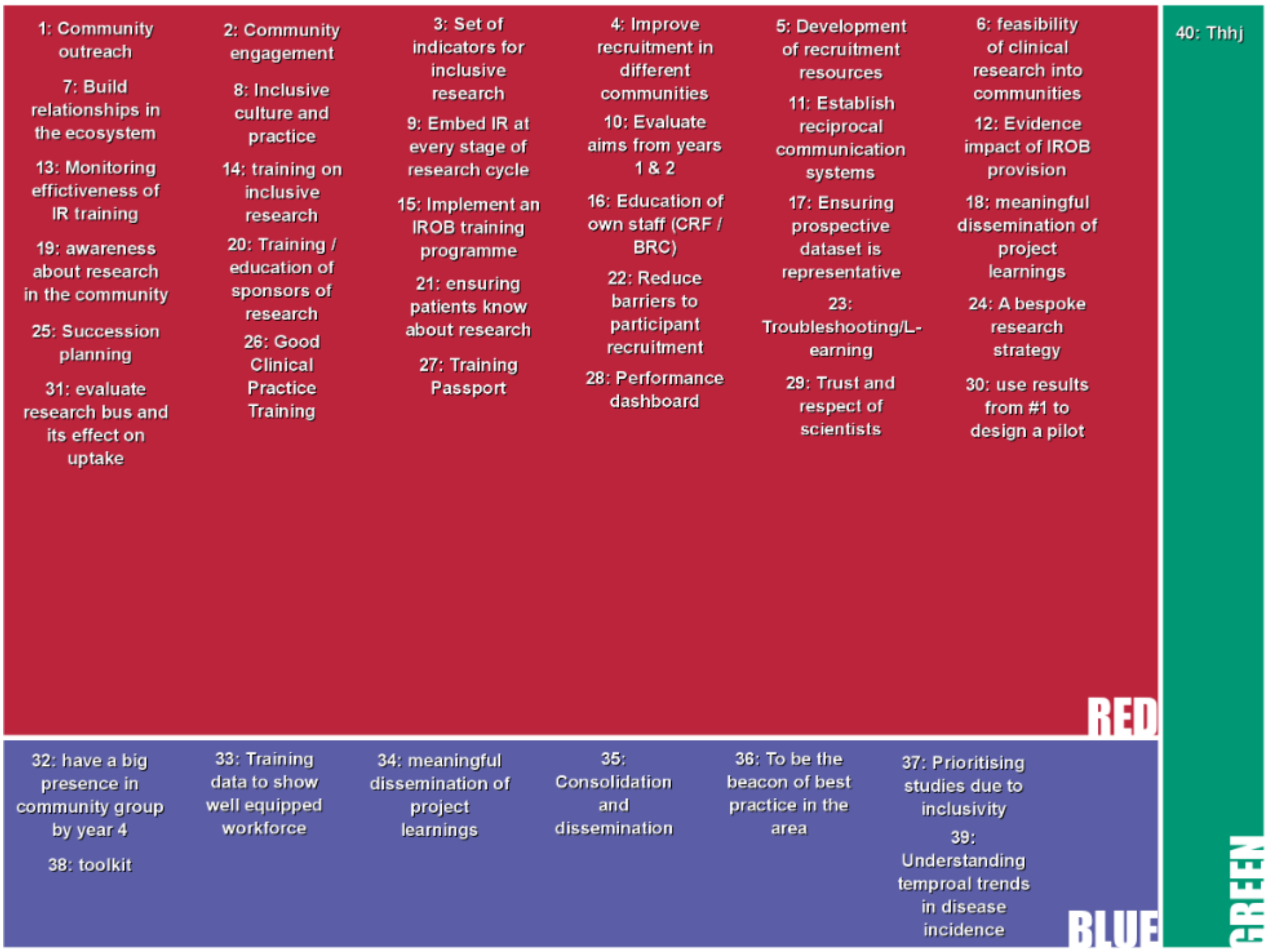
1: Community outreach
7: Build relationships in the ecosystem
13: Monitoring effictiveness of IR training
19: awareness about research in the community
25: Succession planning
31: evaluate research bus and its effect on uptake
2: Community engagement
8: Inclusive culture and practice
14: training on inclusive research
20: Training / education of sponsors of research
26: Good Clinical Practice Training
3: Set of indicators for inclusive research
9: Embed IR at every stage of research cycle
15: Implement an IROB training programme
21: ensuring patients know about research
27: Training Passport
4: Improve recruitment in different communities
10: Evaluate aims from years 1 & 2
16: Education of own staff (CRF / BRC)
22: Reduce barriers to participant recruitment
28: Performance dashboard
5: Development of recruitment resources
11: Establish reciprocal communication systems
17: Ensuring prospective dataset is representative
23: Troubleshooting/L-earning
29: Trust and respect of scientists
6: feasibility of clinical research into communities
12: Evidence impact of IROB provision
18: meaningful dissemination of project learnings
24: A bespoke research strategy
30: use results from #1 to design a pilot
RED
32: have a big presence in community group by year 4
38: toolkit
33: Training data to show well equipped workforce
34: meaningful dissemination of project learnings
35: Consolidation and dissemination
36: To be the beacon of best practice in the area
37: Prioritising studies due to inclusivity
39: Understanding temproal trends in disease incidence
BLUE
40: Thhj
GREEN

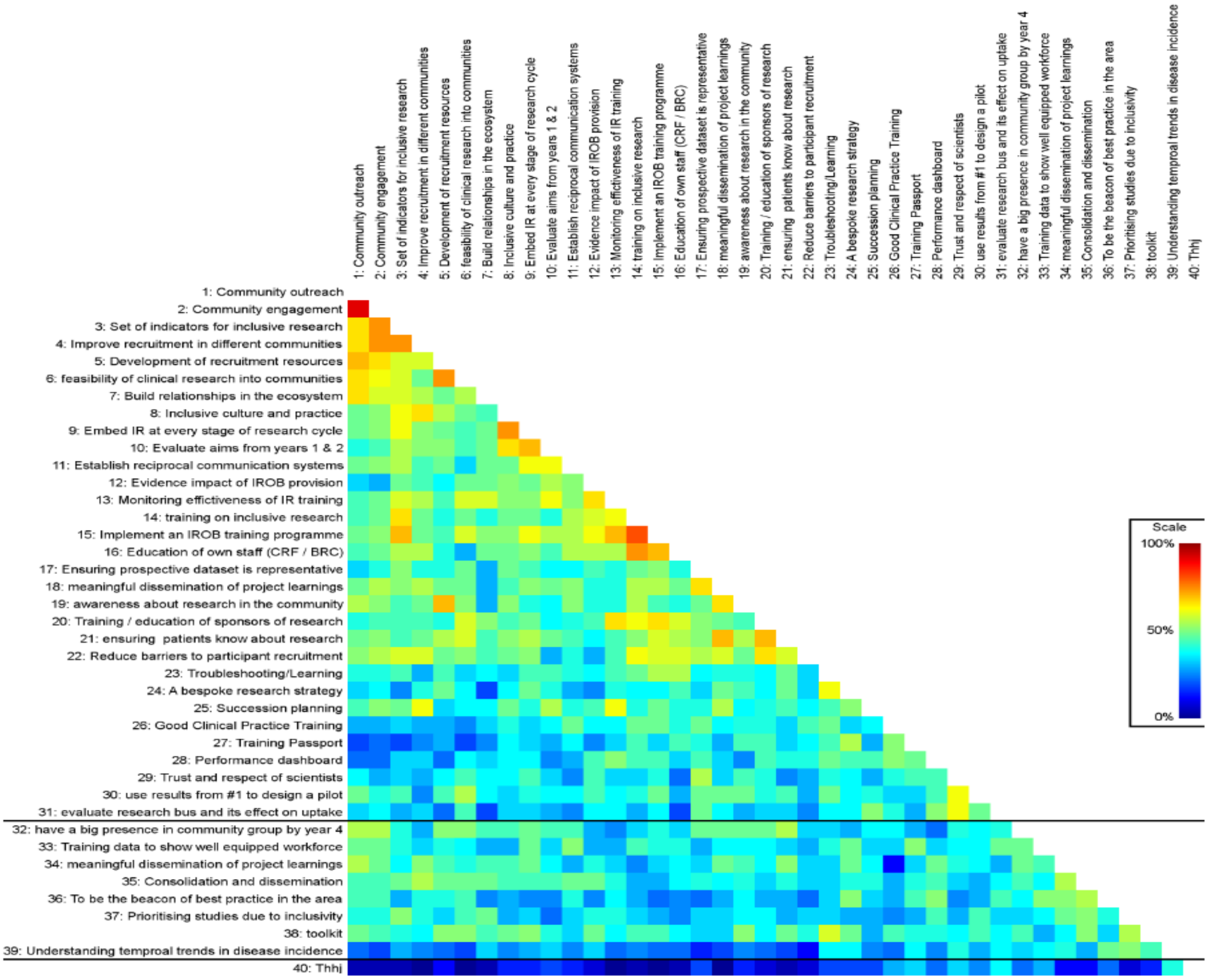
1: Community outreach
2: Community engagement
3: Set of indicators for inclusive research
4: Improve recruitment in different communities
5: Development of recruitment resources
6: feasibility of clinical research into communities
7: Build relationships in the ecosystem
8: Inclusive culture and practice
9: Embed IR at every stage of research cycle
10: Evaluate aims from years 1 & 2
11: Establish reciprocal communication systems
12: Evidence impact of IROB provision
13: Monitoring effectiveness of IR training
14: training on inclusive research
15: Implement an IROB training programme
16: Education of own staff (CRF / BRC)
17: Ensuring prospective dataset is representative
18: meaningful dissemination of project learnings
19: awareness about research in the community
20: Training / education of sponsors of research
21: ensuring patients know about research
22: Reduce barriers to participant recruitment
23: Troubleshooting/Learning
24: A bespoke research strategy
25: Succession planning
26: Good Clinical Practice Training
27: Training Passport
28: Performance dashboard
29: Trust and respect of scientists
30: use results from #1 to design a pilot
31: evaluate research bus and its effect on uptake
32: have a big presence in community group by year 4
33: Training data to show well equipped workforce
34: meaningful dissemination of project learnings
35: Consolidation and dissemination
36: To be the beacon of best practice in the area
37: Prioritising studies due to inclusivity
38: toolkit
39: Understanding temproal trends in disease incidence
40: Thhj
Scale
100%
50%
0%

Q3: How can IROB and the IRT support your plans for inclusive research?

Dendrogram, tree map and heat map created from answers provided by respondents at the workshop

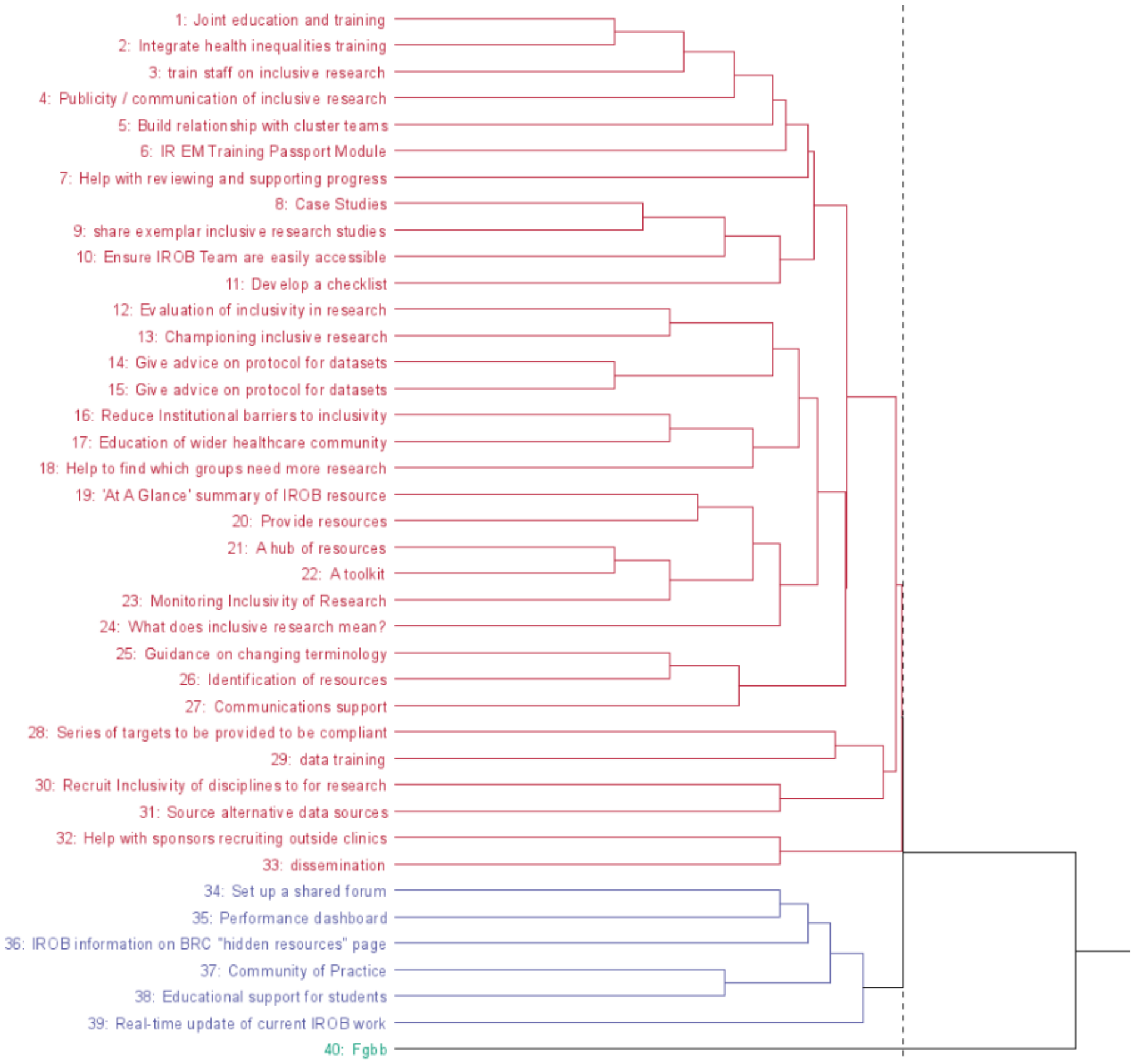

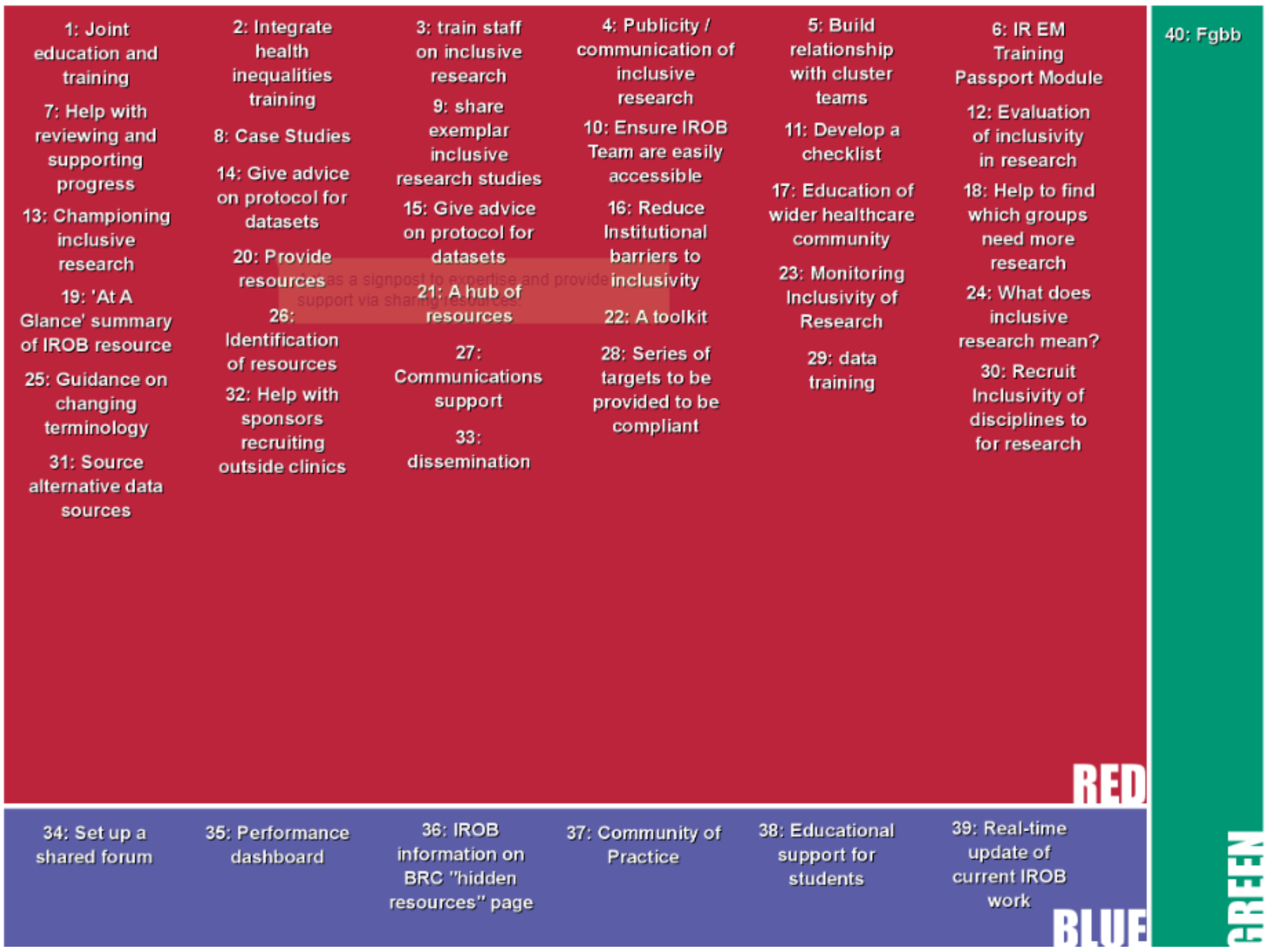
1: Joint education and training
7: Help with reviewing and supporting progress
13: Championing inclusive research
19: 'At A Glance' summary of IROB resource
25: Guidance on changing terminology
31: Source alternative data sources
2: Integrate health inequalities training
8: Case Studies
14: Give advice on protocol for datasets
20: Provide resources
26: Identification of resources
32: Help with sponsors recruiting outside clinics
3: train staff on inclusive research
9: share exemplar inclusive research studies
15: Give advice on protocol for datasets
21: A hub of resources
27: Communications support
33: dissemination
4: Publicity / communication of inclusive research
10: Ensure IROB Team are easily accessible
16: Reduce Institutional barriers to inclusivity
22: A toolkit
28: Series of targets to be provided to be compliant
5: Build relationship with cluster teams
11: Develop a checklist
17: Education of wider healthcare community
23: Monitoring Inclusivity of Research
29: data training
6: IR EM Training Passport Module
12: Evaluation of inclusivity in research
18: Help to find which groups need more research
24: What does inclusive research mean?
30: Recruit Inclusivity of disciplines to for research
RED
34: Set up a shared forum
35: Performance dashboard
36: IROB information on BRC "hidden resources" page
37: Community of Practice
38: Educational support for students
39: Real-time update of current IROB work
BLUE
40: Fgbb
GREEN

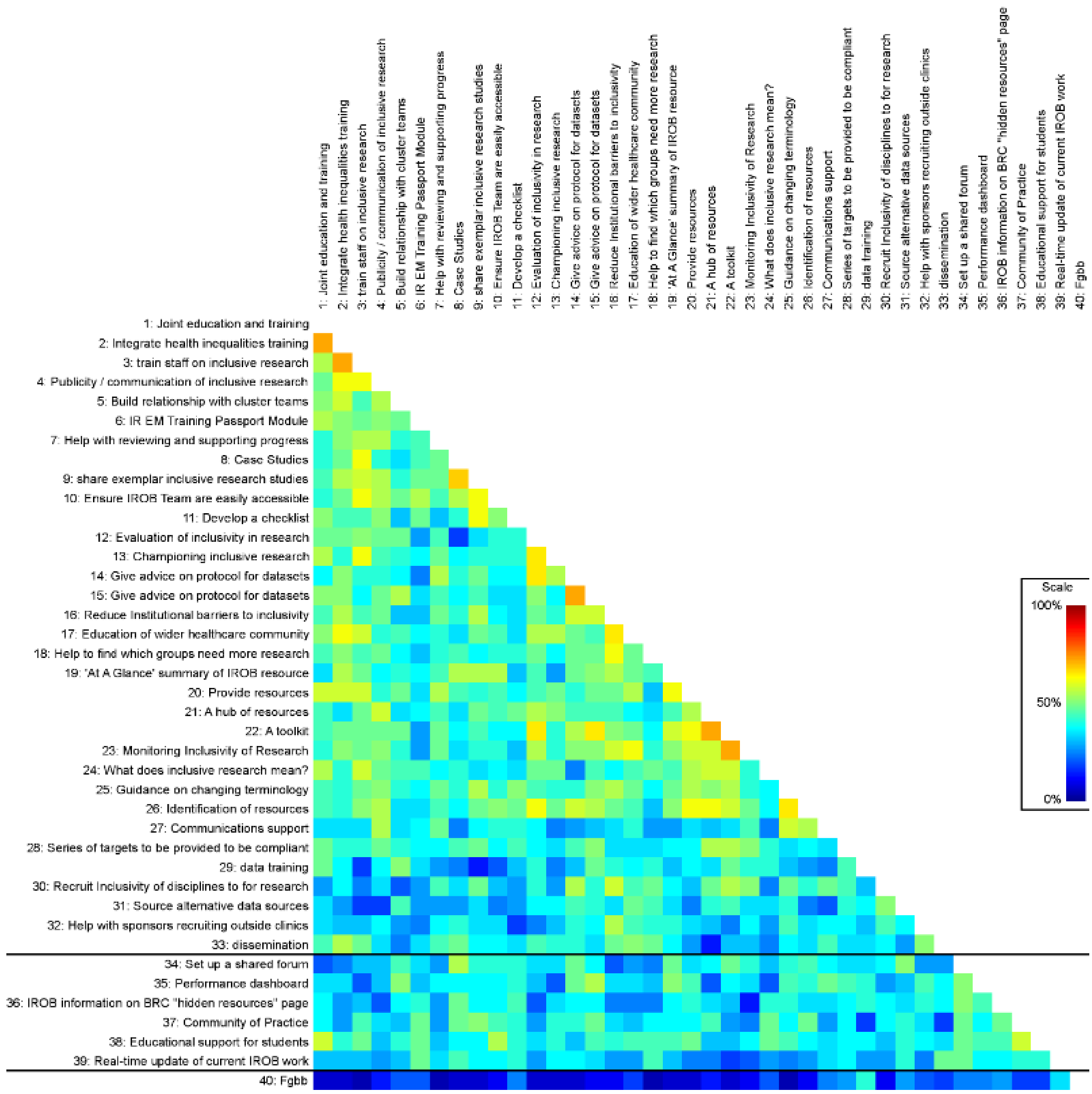
1: Joint education and training
2: Integrate health inequalities training
3: train staff on inclusive research
4: Publicity / communication of inclusive research
5: Build relationship with cluster teams
6: IR EM Training Passport Module
7: Help with reviewing and supporting progress
8: Case Studies
9: share exemplar inclusive research studies
10: Ensure IROB Team are easily accessible
11: Develop a checklist
12: Evaluation of inclusivity in research
13: Championing inclusive research
14: Give advice on protocol for datasets
15: Give advice on protocol for datasets
16: Reduce Institutional barriers to inclusivity
17: Education of wider healthcare community
18: Help to find which groups need more research
19: 'At A Glance' summary of IROB resource
20: Provide resources
21: A hub of resources
22: A toolkit
23: Monitoring Inclusivity of Research
24: What does inclusive research mean?
25: Guidance on changing terminology
26: Identification of resources
27: Communications support
28: Series of targets to be provided to be compliant
29: data training
30: Recruit Inclusivity of disciplines to for research
31: Source alternative data sources
32: Help with sponsors recruiting outside clinics
33: dissemination
34: Set up a shared forum
35: Performance dashboard
36: IROB information on BRC "hidden resources" page
37: Community of Practice
38: Educational support for students
39: Real-time update of current IROB work
40: Fgbb
Scale
100%
50%
0%

Q4: How can IROB and the IRT support with your participatory plans?

Dendrogram, tree map and heat map created from answers provided by respondents at the workshop

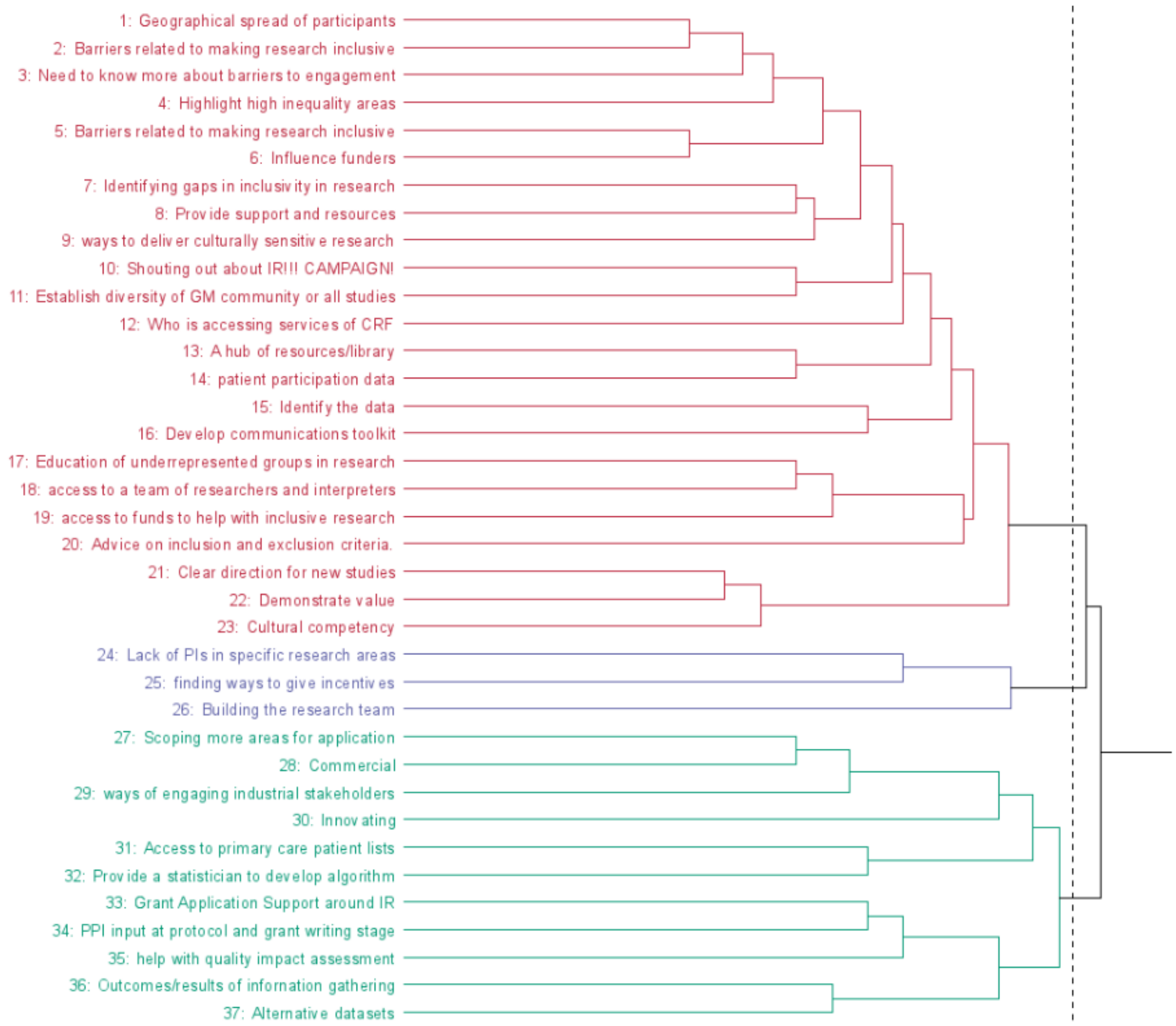

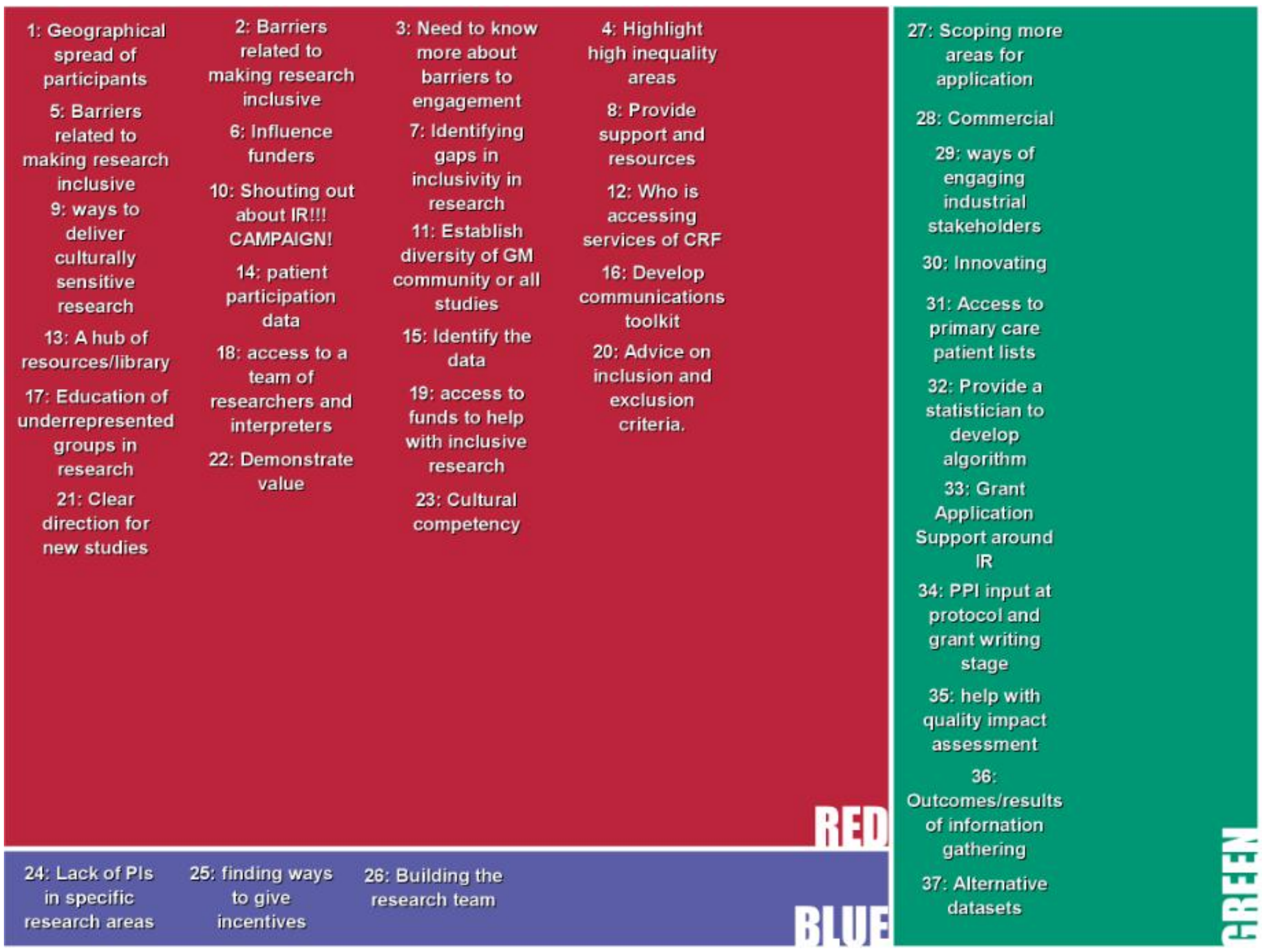

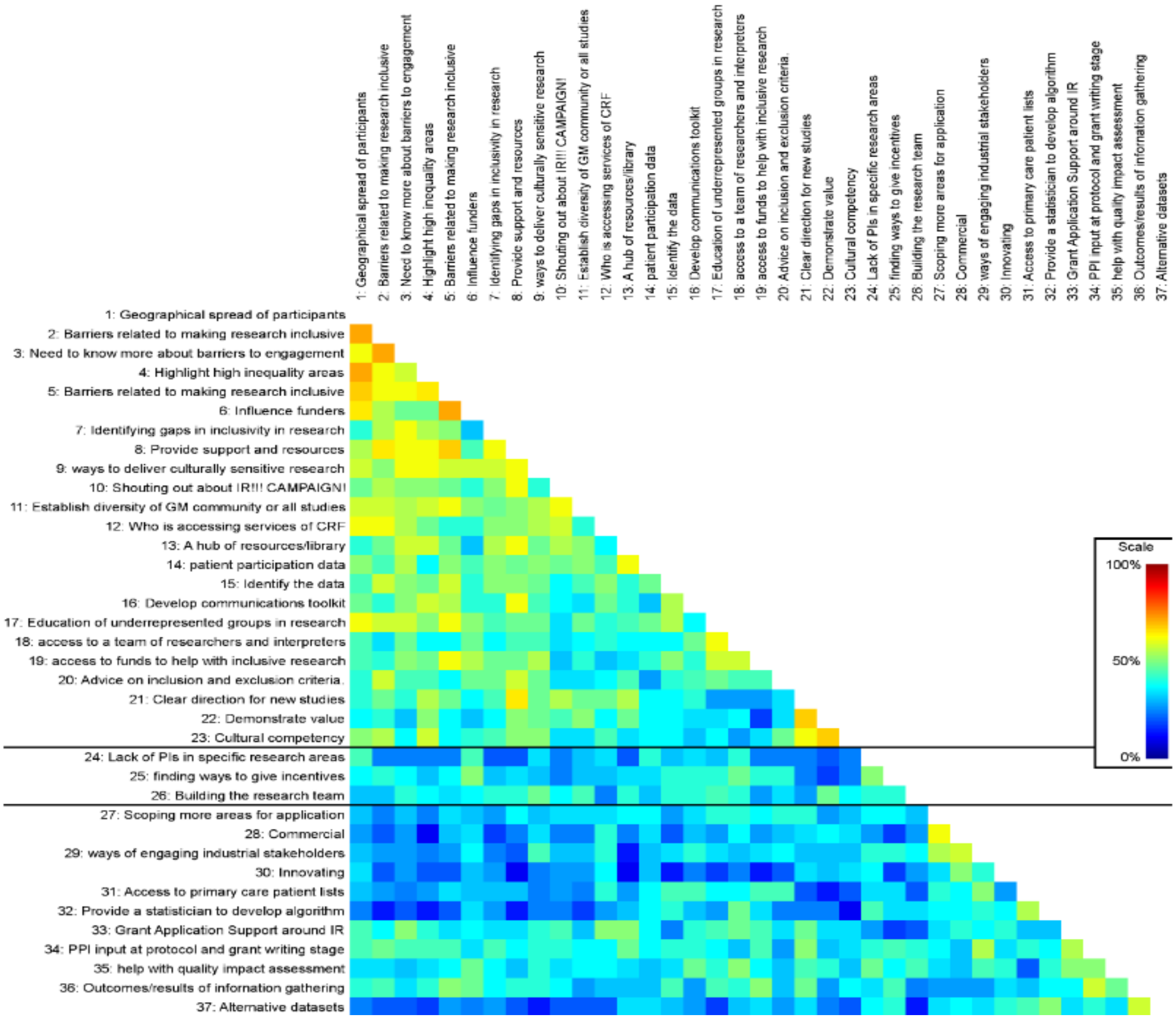
1: Geographical spread of participants
2: Barriers related to making research inclusive
3: Need to know more about barriers to engagement
4: Highlight high inequality areas
5: Barriers related to making research inclusive
6: Influence funders
7: Identifying gaps in inclusivity in research
8: Provide support and resources
9: ways to deliver culturally sensitive research
10: Shouting out about IR!!! CAMPAIGN!
11: Establish diversity of GM community or all studies
12: Who is accessing services of CRF
13: A hub of resources/library
14: patient participation data
15: Identify the data
16: Develop communications toolkit
17: Education of underrepresented groups in research
18: access to a team of researchers and interpreters
19: access to funds to help with inclusive research
20: Advice on inclusion and exclusion criteria.
21: Clear direction for new studies
22: Demonstrate value
23: Cultural competency
24: Lack of PIs in specific research areas
25: finding ways to give incentives
26: Building the research team
27: Scoping more areas for application
28: Commercial
29: ways of engaging industrial stakeholders
30: Innovating
31: Access to primary care patient lists
32: Provide a statistician to develop algorithm
33: Grant Application Support around IR
34: PPI input at protocol and grant writing stage
35: help with quality impact assessment
36: Outcomes/results of information gathering
37: Alternative datasets
Scale
100%
50%
0%
